# Unlocking biological insights from differentially expressed genes: Concepts, methods, and future perspectives

**DOI:** 10.1016/j.jare.2024.12.004

**Published:** 2024-12-06

**Authors:** Huachun Yin, Hongrui Duo, Song Li, Dan Qin, Lingling Xie, Yingxue Xiao, Jing Sun, Jingxin Tao, Xiaoxi Zhang, Yinghong Li, Yue Zou, Qingxia Yang, Xian Yang, Youjin Hao, Bo Li

**Affiliations:** a College of Life Sciences, Chongqing Normal University, Chongqing 401331, PR China; b Department of Neurosurgery, Xinqiao Hospital, The Army Medical University, Chongqing 400037, PR China; c Department of Neurobiology, Chongqing Key Laboratory of Neurobiology, The Army Medical University, Chongqing 400038, PR China; d Department of Biology, College of Science, Northeastern University, Boston, MA 02115, USA; e Chongqing Key Laboratory of Big Data for Bio Intelligence, Chongqing University of Posts and Telecommunications, Chongqing 400065, PR China; f Zhejiang Provincial Key Laboratory of Precision Diagnosis and Therapy for Major Gynecological Diseases, Women’s Hospital, Zhejiang University School of Medicine, Hangzhou 310058, PR China

**Keywords:** Differentially expressed genes, Molecular mechanisms, Analytical strategies, Biological interpretation, Biological process

## Abstract

**Background:**

Identifying differentially expressed genes (DEGs) is a core task of transcriptome analysis, as DEGs can reveal the molecular mechanisms underlying biological processes. However, interpreting the biological significance of large DEG lists is challenging. Currently, gene ontology, pathway enrichment and protein–protein interaction analysis are common strategies employed by biologists. Additionally, emerging analytical strategies/approaches (such as network module analysis, knowledge graph, drug repurposing, cell marker discovery, trajectory analysis, and cell communication analysis) have been proposed. Despite these advances, comprehensive guidelines for systematically and thoroughly mining the biological information within DEGs remain lacking.

**Aim of review:**

This review aims to provide an overview of essential concepts and methodologies for the biological interpretation of DEGs, enhancing the contextual understanding. It also addresses the current limitations and future perspectives of these approaches, highlighting their broad applications in deciphering the molecular mechanism of complex diseases and phenotypes. To assist users in extracting insights from extensive datasets, especially various DEG lists, we developed DEGMiner (https://www.ciblab.net/DEGMiner/), which integrates over 300 easily accessible databases and tools.

**Key scientific concepts of review:**

This review offers strong support and guidance for exploring DEGs, and also will accelerate the discovery of hidden biological insights within genomes.

## Highlights

• The first review to systematically introduce and summarize the tools for maximizing biological information of genes.
• A comprehensive overview of representative tools and algorithms for analyzing differentially expressed genes.
• More than 300 tools, databases, and algorithms are summarized on the website DEGMiner.
• A detailed guideline is provided to help researchers better mine the functions and interactions of genes.

## Graphical abstract

**Figure:**
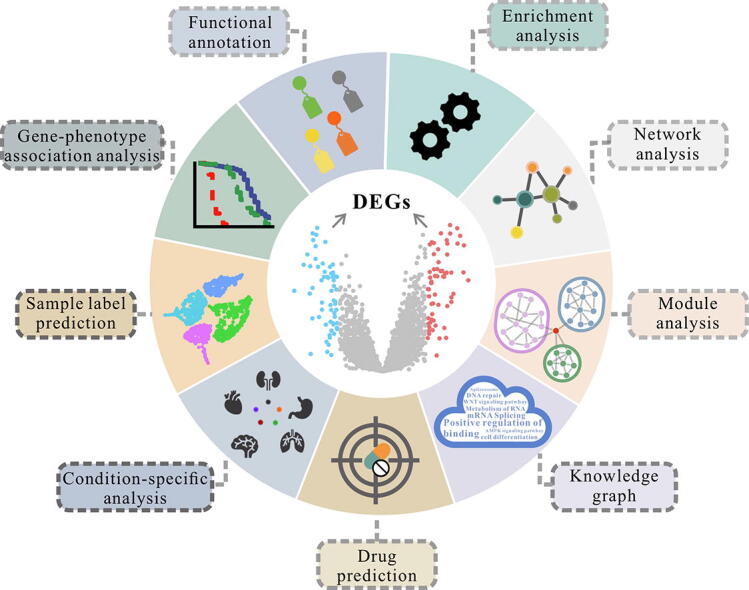

## Introduction

Transcriptome analysis plays a crucial role in determining the gene expression changes among individuals with and/or without specific diseases. This analysis helps identify differentially expressed genes (DEGs) that may be linked to the investigated disease as genetic triggers, consequences, or indicators [1]. In biomedicine, computationally exploring DEGs has become an essential strategy, aiding in the unraveling of the underlying mechanisms behind complex diseases such as cancer [2], [3], Alzheimer’s disease [4], and epilepsy [5], or in elucidating the body’s response to drug stimulation [6], aging [7], and other perturbations [8].

Among the approaches used for analyzing and mining DEGs, Gene Ontology (GO) and Kyoto Encyclopedia of Genes and Genomes (KEGG) are widely considered as the two most significant ones [9], [10]. GO focuses on the basic biological functions of genes, and KEGG emphasizes the genes within pathways. Nevertheless, both methods share a common flaw of overlooking the interactions between genes or their products [11], [12]. To conquer this limitation, protein–protein interaction (PPI) networks and gene regulatory networks (GRNs) have been employed to explore the interactions among DEGs. Unfortunately, PPI and GRN approaches often neglect the function of non-coding genes (*e.g.*, microRNA) and metabolites that play significant roles in complex biological processes [13], [14], [15]. To bridge these gaps, emerging approaches such as module/pattern analysis, bio-network analysis, survival analysis, drug repurposing, and knowledge graphs (KGs) have been proposed. These approaches provide tailored analyses to investigate diverse states and conditions that regulate biological responses to specific perturbations. For instance, weighted gene co-expression network analysis (WGCNA) has been successfully applied to identify modules associated with autism spectrum disorder [16]. A growing number of omics studies have demonstrated their importance in identifying gene dysfunction in diseases [17], [18] and determining the impact of therapeutic intervention on biological responses [19].

An important task for biologists working in the biomedicine is to understand cellular functions, which requires in-depth interpretation of information from DEGs and building accurate cellular models to generate test hypotheses. Despite advances in methodologies, databases and tools, some researchers still face challenges in fully understanding the specialized bioinformatics techniques for biological interpretation. So far, the scientific community has not reached a consensus on how to comprehensively analyze DEGs to extract effective biological information.

In this systematic review, we summarize popular approaches and provide practical guidelines for deciphering DEGs to uncover the molecular mechanisms underlying gene function. These approaches encompass gene annotation, gene set enrichment analysis, gene-associated networks [including gene regulatory, competing endogenous RNA (ceRNA), and PPI networks], module/pattern analysis (such as co-expression, co-regulation, and other modules), KGs, drug repurposing, clustering analysis, and more. We have compiled numerous related databases and tools on a website named DEGMiner (https://www.ciblab.net/DEGMiner/), which offers convenient access to over 300 databases and tools. To the best of our knowledge, this is the first comprehensive review on deeply mining DEG information across various groups or conditions. It serves as a valuable reference for researchers in the biological field, enabling deeper exploration of DEGs and facilitating the discovery of molecular mechanisms underlying complex diseases, phenotypes, and biological behaviors.

## Analytical strategies for deciphering the biological significance of DEGs

### Accurate functional annotation of genes

#### Identification of orthologous genes across species

Functional annotation and enrichment analysis are the pivotal initial steps in speculating gene functions. Existing bioinformatics tools for predicting gene function primarily cater to model organisms, such as *Homo sapiens* and *Mus musculus*, limiting their applicability to non-model species. Specially, this constraint poses significant challenges when analyzing gene function for non-model organisms. Even in medical research involving model organisms like *Mus musculus*, it is still crucial to identify homologous genes playing key functionalities in humans, especially in drug development. Therefore, accurately translating genes from model/non-model species into homologs (*i.e.*, orthologous genes) of the common model species is a necessary step before utilizing existing sophisticated tools for gene function analysis. Current ortholog conversion tools fall into two categories: sequence-free and sequence-based methods. Sequence-free tools convert gene Entrez identifiers (IDs) or symbols directly between diverse species, with examples including AllEnricher [20], biomaRt [21], homologene, OMA browser [22], gprofiler2 [23] and KEGG Orthology. While this direct conversion works well for model species, it can be awkward for non-model species where gene Entrez IDs or symbols are not readily available for downstream analysis. Complementary to sequence-free tools, sequence-based methods identify orthologous genes through DNA sequence comparison, effectively overcoming this limitation. Notable examples of such tools and databases include OMA browser, eggNOG [24], OrthoVenn2 [25], OrthoDB [26] and ORCAN [27].

#### Gene ontology-based annotation

GO is a standardized framework designed to describe the functional attributes of gene products, containing molecular function (MF), biological process (BP) and cellular components (CC). GO facilitates the integration of annotations from diverse databases, providing a unified approach to characterize gene function [28]. Numerous bioinformatics tools have been developed to annotate and visualize gene functions for DEG lists according to GO terms, and these tools primarily vary in their breadth, scope, and depth of annotation. For instance, WEGO is a tool tailored for GO annotation and visualization of large-scale genomic data across nine model organisms [29]. In contrast, g:Profiler broadens the scope of gene annotation by offering an extensive array of gene and protein functional annotation tools, including GO, pathways, and disease associations [30]. Similarly, agriGO specializes in the annotation of plant gene ontology [31]. Instead of using gene lists, Blast2GO is one of the widely used tools for assigning GO terms to sequences based on similarity search, providing advanced functional analysis in research of non-model species and demonstrating powerful capability of annotating large high-throughput sequences derived from transcriptomics and metagenomics [32].

The standard semantic system provided by GO offers significant advantages in advancing the research of gene functions. However, they also present some limitations. One major limitation is the potential for these standard descriptions to be overly broad and general, which may lead to a lack of detailed descriptions for unique gene functions within specific biological contexts. Consequently, it is imperative for researchers to integrate contextual and detailed information to fully comprehend the specific functions and mechanisms of genes, when utilizing GO for gene function analysis. This approach helps avoid an overreliance on standardized descriptions that might overlook the distinct characteristics of individual genes.

#### Biological pathway annotation

Although GO annotation provides a lot of information about the basic functions of genes, its terms can be too broad and generic, which may obscure specific and nuanced functions of individual genes within particular biological contexts. To fill these gaps, signaling pathways provide a more detailed description of gene function by illustrating how biomolecules cooperate to carry out cellular tasks in various conditions [33].

**Fig. 1:**
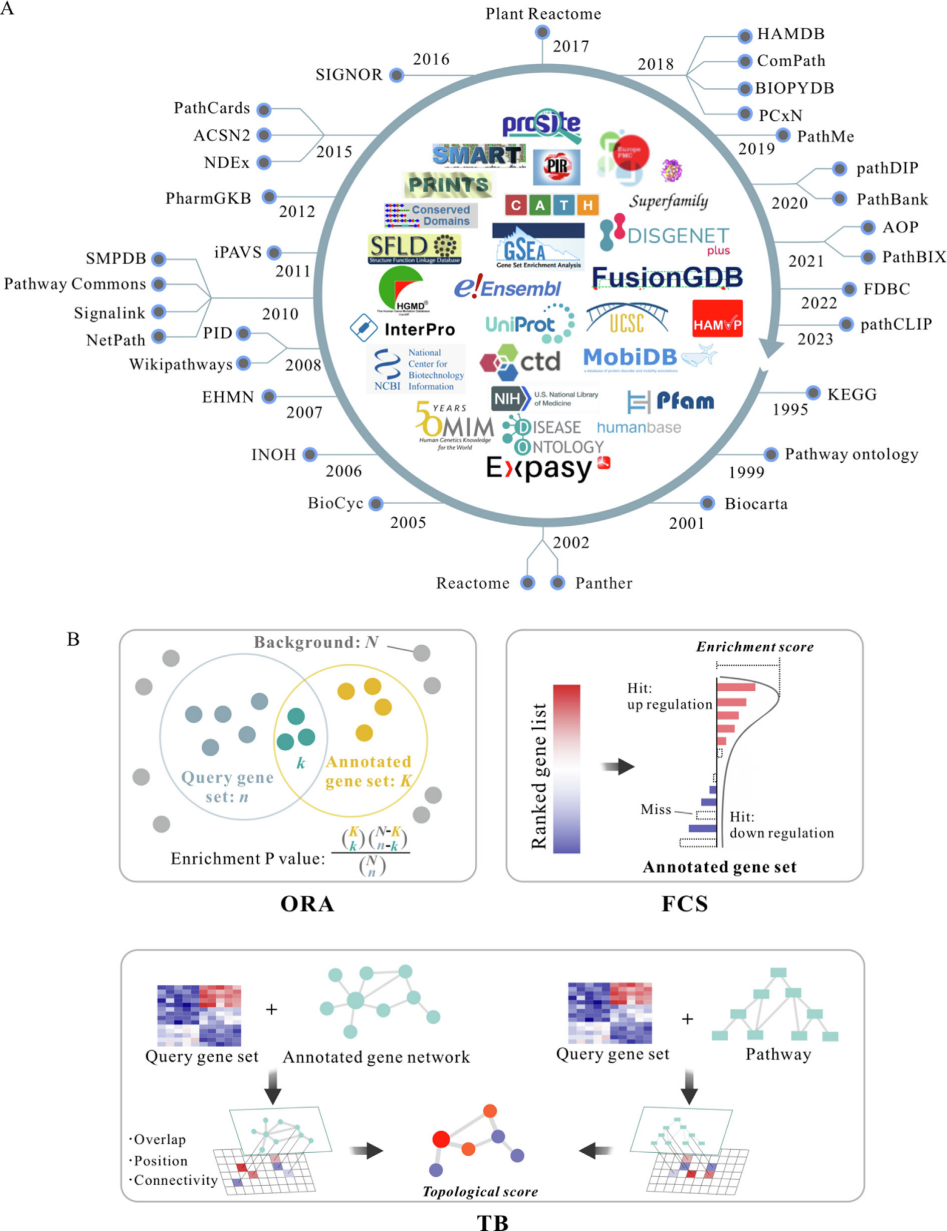
Overview of gene functional annotation databases and enrichment analysis methodologies. A) Some representative pathway databases and gene functional annotation databases. It is noteworthy that 13 of the aforementioned databases are members of the InterPro Consortium, which include CATH-Gene3D, CDD, HAMAP, MobiDB, PANTHER, Pfam, PIRSF, PRINTS, PROSITE, SFLD, SMART, SUPERFAMILY, and NCBIfam. B) Three types of methods for enrichment analysis. The schematic diagram demonstrates that ORA employs a hypergeometric test to assess whether the number of query genes is significantly higher than expected by chance. FCS method ranks the gene set based on gene expression levels and tests if the hit genes map to the annotated gene set. TB method integrates scores that measure genes' connectivity within the expression level and their position within a network.

The pathway is a collection of physically interacting molecules, primarily proteins and metabolites, organized in specific directions with defined upstream and downstream relationships. To fully understand the biological roles these molecules play in various life processes, it is essential to annotate pathways within their broader context, taking into account their interactions and functions from a holistic perspective. Till now, at least 30 pathway-related databases have been developed, as shown in Fig. 1**A**. Over the past three decades, the pathway databases have evolved in four main directions: (1) Species-specific databases, such as FDBC for fungal-related pathways and Plant Reactome for plant-related pathways; (2) The databases emphasizing the function-specific pathways, exemplified by the Human Autophagy Database (HAMDB), which consolidates information on cell autophagy; (3) Interactive pathway visualization tools, such as Reactome and the Small Molecule Pathway Database (SMPDB); and (4) The comprehensive databases that also exhibit particular strengths in specific areas, such as KEGG with its diverse datasets (genomics, metabolic pathways, and diseases), PathBank with its unique collection of pathways (covering over 100,000 pathways), and Wikipathways characterized by its openness. Based on those distinct characteristics of pathway databases, it is highly worthwhile to obtain further functional analysis through pathway annotation for very small and gene-dispersed DEG sets across different pathways.

#### Other annotations of genes

Beyond the conventional GO and KEGG pathway annotations, there are many valuable annotations, such as chromosome cytobands or subcellular gene locations, which provide deeper insights into the intricate correlation between molecular mechanisms and phenotypic traits. Additionally, other types of annotations also include terms related to disease-associated genes, human/mouse phenotypes, hallmark genes, oncogenic signatures, immunologic signatures, cell type signatures, developmental stages, gene variants, literature references, and protein domains.

These diverse annotation strategies have been recognized by tool developers, who have integrated them into various platforms to enhance analytical power. As illustrated in Fig. 1**A** and detailed in Supplementary Material Table S1, comprehensive tools like Gene Set Enrichment Analysis (GSEA [34]) encompass the majority of annotation types mentioned previously, making them widely applicable across multiple research areas. Besides, some tools specialize in specific annotation types. For instance, the Disease Ontology [35], which focuses on disease-associated genes, has gained significant popularity for its relevance in disease research.

### Enrichment analysis

Enrichment analysis offers a distinct approach to computationally identify the biologically significant patterns within a given set of features, such as DEGs concentrated in this review. Rather than mapping genes to their biological annotations directly, enrichment analysis compares the distribution of terms within a gene set of interest against a background distribution to statistically determine the likelihood of a nonrandom distribution [36]. Enrichment analysis can be carried out against various annotation items, such as gene ontology and biological pathways, making it a prominent computational method for integrating newly identified DEGs into existing biological knowledge [34].

Enrichment analysis tools are categorized into three main types according to mathematical principles: over-representation analysis (ORA), functional class scoring (FCS), and topology-based (TB) methods (Fig. 1**B**). ORA is one of the simplest methods, identifying whether annotated gene sets are over-represented within a given gene set. Tools like DAVID [36] and ToppGene [37] typically use hypergeometric or Fisher's exact tests for ORA to assess the significance of gene set enrichment compared to random chance. Regrettably, ORA overlooks gene-gene relationships and relies heavily on thresholds, which can result in the loss of important information and reduced reliability and reproducibility. Additionally, the use of inappropriate background gene sets and outdated annotation databases can significantly affect ORA results [38]. To overcome these limitations, the FCS and TB methods were proposed, which reduce reliance on background gene sets. FCS, a threshold-free method, uses all gene expression values (including those with small changes in magnitude) to rank genes and then generates a gene set score that reflects the level of enrichment of genes within the set. GSEA, a representative example of FCS [34], ranks genes based on their differential expression levels and calculates an enrichment score for specific gene sets, indicating activation or inhibition based on their position in the ranked list. The TB methods integrate gene expression data with the topological structure of genes and calculate their position, connectivity, and overlap with other gene sets within the network and pathway. Sequentially, they evaluate the association of the gene set with specific biological processes or pathways by comparing the characteristics with those of other gene sets in the entire network. This assessment is based on the observed differences between the characteristics of the target gene set and others within the network [39], such as Pathway-Express [40] and SPIA [41]. However, the computational complexity and the requirement for precise relationships in the input data make these methods costly to compute and the results more complex to interpret.

Enrichment analysis is widely used in omics research to characterize the holistic function of a given gene set. Since omics datasets from proteomics, single-cell RNA sequencing (scRNA-seq), genome-wide association studies (GWAS) and epigenomics exhibit different statistical distributions compared to bulk gene expression data [38], the adaptability of enrichment methods to these varying distributions must be fully considered during data analysis. For example, in proteomics, where detection is biased towards highly expressed proteins and quantifying protein complexes is challenging, FCS-based tools can help mitigate variability and identify robustly enriched protein sets. Numerous enrichment tools have been developed based on these methods, as detailed in Supplementary Material Table S1. As yet, there is insufficient evidence to definitively recommend specific methods for each type of omics. Given the distinct characteristics of each method, using both ORA and FCS/TB methods together may provide more comprehensive and reliable results for identifying enriched pathways or functional categories. Further research and comparative studies are necessary to validate this hypothesis and determine optimal enrichment strategies across different omics datasets. An updated list of web-based tools and R/Python packages is provided in Table 1.

**Table 1:** Some representative software for gene set enrichment analysis.

**Name**	**Statistical method**	**Resource**	**PMID**
DAVID	Fisher's exact test (modified as EASE score)	https://david.ncifcrf.gov	19131956
GSEA	Kolmogorov-Smirnov-like test	https://www.gsea-msigdb.org	16199517
clusterProfiler	Hypergeometric test	Bioconductor package	22455463
Enrichr	Fisher’s exact test	https://maayanlab.cloud/Enrichr/	27141961
PANTHER	Binomial test, Fisher's exact test	https://pantherdb.org	12952881
ClueGO	Hypergeometric test	Cytoscape plugin	19237447
Toppgene	Fisher’s inverse χ^2^ test, hypergeometric test	https://toppgene.cchmc.org	19465376
EnrichmentMap	Fisher’s exact test	Cytoscape plugin	21085593
Metascape	Hypergeometric test	https://metascape.org/	30944313
g:Profiler	Hypergeometric test	https://biit.cs.ut.ee/gprofiler/ and CRAN package	27098042
GAGE	Meta-test	Bioconductor package	19473525
PAGE	Z-score	Python module	15941488
GeneCodis	Hypergeometric test, χ^2^ test	https://genecodis.genyo.es/	17204154
iDEP	Student’s *t*-test	https://ge-lab.org/idep/	30567491
hypeR	Hypergeometric test	https://github.com/montilab/hypeR	31498385

### Gene-associated network analyses

Genes are often functionally interdependent to maintain intercellular and intracellular homeostasis, organizing a complex network with genetic interactions. It is reported that many complicated diseases are caused by perturbated gene networks rather than a single genetic abnormality [42]. Therefore, it is necessary to unravel the regulatory relationships among genes through network analyses, which generally require a holistic exploration and interpretation from different genomic perspectives. At the transcriptome level, genes are directly or indirectly regulated by transcription factors (TFs), microRNAs (miRNAs), and long non-coding RNAs (lncRNAs) [13], [43], forming a variety of gene-related networks (including gene regulation, TF regulation, miRNA regulation, and ceRNA networks). At the protein level, PPI reflects direct physical interactions, helping understand protein functions, identify drug targets, reveal disease mechanisms, and support systems biology research [44]. Collectively, gene-associated network analyses are of vital importance in predicting gene functions, identifying regulation modules related to diseases, and guiding drug design and screening. Table 2 and Supplementary Material Table S2 summarize current knowledge about gene-associated networks, relevant databases, and popular tools for network analysis and visualization.

**Table 2:** The tools used for network analysis and visualization.

**Name**	**gene-gene**	**gene → gene**	**TF → gene**	**miRNA → gene**	**Year of Release**	**PMID**
Cytoscape	✓	✓	✓	✓	2003	14597658
GeneMANIA	✓	✗	✓	✓	2018	29912392
ConsensusPathDB	✓	✓	✗	✗	2016	27606777
iRegulon	✓	✓	✓	✓	2014	25058159
NetworkAnalyst	✓	✓	✓	✓	2014	24861621
NAViGaTOR	✓	✓	✗	✗	2009	19837718
BioProfiling.de	✓	✗	✗	✓	2011	21609949
FunCoup	✓	✗	✓	✓	2009	19246318
miRTargetLink	✗	✗	✗	✓	2016	27089332
SNOW	✓	✗	✗	✗	2009	19454602
FFLtool	✗	✗	✓	✓	2020	31830251

#### Gene regulatory network analysis

**Fig. 2:**
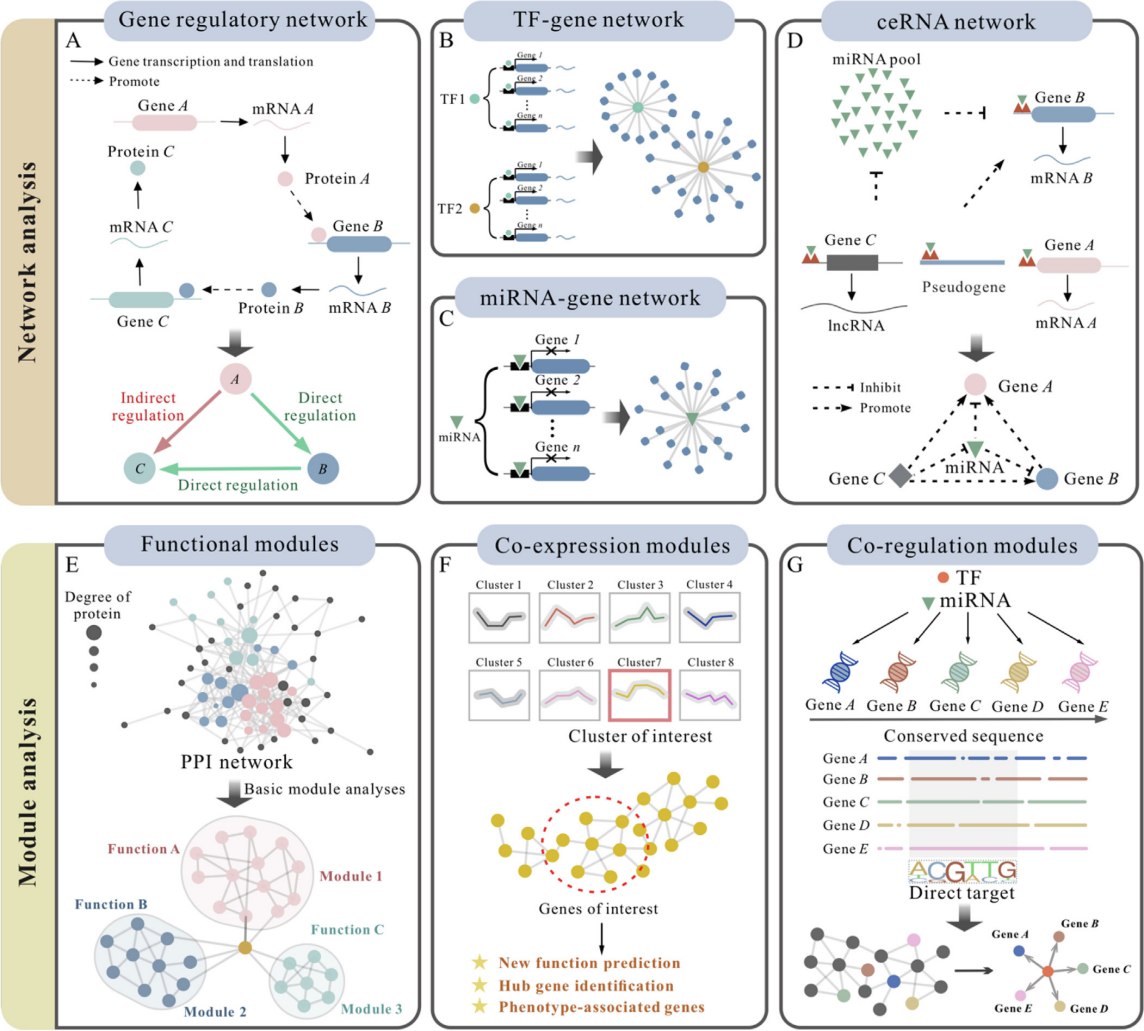
Gene-associated networks and modules schematic. **A)** Gene regulatory network. **B-C)** TF-gene and miRNA-gene interaction networks. The green triangle, circle with two colors and blue cylinder stand for the miRNA, TF and mRNA, respectively. **D)** The ceRNA network. The triangle, circle and rhombus stand for the miRNA, mRNA and lncRNA, respectively. Gene *B* interacts with miRNA through miRNA response element (MRE) binding sites, leading to the inhibition of its expression by miRNA binding. miRNA can also be regulated by interacting with Gene *C* (lncRNA), pseudogenes or Gene *A*, by MRE site interaction with their seed sequence. ceRNA crosstalk is influenced by the expression levels of all RNA molecules participating in the network, behaving as “competitors” for the same miRNA cluster. **E)** Protein-protein interaction network and the related functional modules. In PPI network, the nodes indicate the proteins, with the size of the node (degree) indicating the number of links to a given node. Different colors represent the different functions of proteins. **F)** Co-expression modules. **G)** Co-regulation modules.

Gene regulatory network (GRN) is one of the fundamental structures describing gene expression patterns, consisting of both direct and indirect regulatory interactions [45], [46], [47]. Indirect regulatory interactions occur when a gene (*e.g.*, gene *A*) regulates the expression of gene *B*, which in turn regulates the expression of gene *C* (Fig. 2**A**). In this scenario, gene *A* and *B* are considered to have a direct regulatory interaction, while gene *A* and *C* exhibit an indirect regulatory interaction. GRNs have emerged as powerful tools for capturing gene-gene interactions that govern mRNA and protein expression levels, thus providing deep outlooks into the complex landscape of transcriptional regulation. Constructing a GRN involves leveraging existing knowledge and customizing it to a specific biological context. The information for building GRNs is typically collected manually from empirical research studies and curated in dedicated knowledge bases. These databases for constructing GRNs fall into three categories: interactome databases (*e.g.*, IntAct [48], SIGNOR [49], BIND [50], PID [51] and Spike [52]), specific function-related databases (*e.g.*, InnateDB [53]) and comprehensive open databases (*e.g.*, NDEx [54]).

#### TF-regulation network

The use of higher-order regulatory patterns is also particularly important for gene regulation. The regulators, known as transcription factors (TFs), are proteins that bind to either enhancer or promoter regions of DNA adjacent to the target genes and determine the on/off state of genes. The interaction between several TFs and their target genes forms a complex TF-regulation network in cells (Fig. 2**B**), and it may be closely associated with many pathological processes, including cellular malfunction and disease pathways [55], and so on. For instance, Rauch *et al.* identified a diverse transcriptional network comprising pro-osteogenic and antiadipogenic TFs through analyzing the interactions between DEGs and TFs, which sheds new light on disease therapy [56]. TF-gene networks provide valuable knowledge about disease mechanisms and the impact of critical genes on diseases, thereby offering potential clues for clinical diagnosis.

Several databases currently compile the detailed information on TF-regulation network, and different databases with varying preferences and objectives bring forth distinct emphases. AnimalTFDB [57], functioning as a repository for animal TFs information, aids research concerning animals. In contrast, investigations into TF regulatory networks in plants or fungi can utilize JASPAR [58], which predicts TF-gene binding sites, and ENCODE [59], which furnishes extensive gene regulatory information. In the field of plant research, PlantCARE demonstrates a useful web tool, with the powerful capability of predicting the regulatory relationships between TFs and *cis*-acting elements from a set of co-regulated genes [60]. Naturally, for specific diseases, databases such as CHEA [61], focusing on TF-gene relationships in disease progression, are more suitable. Furthermore, comprehensive GRN maps provided by databases like RegNetwork [62] facilitate the comprehension of the intricacies and dynamics inherent in GRNs.

#### miRNA-regulation network

MicroRNAs (*i.e.*, miRNAs) have arisen as another class of transcription regulator component which have huge effects on the expression levels of genes [63]. The miRNAs bind to the 3′ untranslated region or other regions of target genes, leading to the silencing or degradation of the target genes. Those silencing interactions are of great importance in many biological processes, including cell development, differentiation, and homeostasis [64], [65]. Similar to TF-regulation networks, miRNA-regulation networks are created in a combinatorial manner (Fig. 2**C**), which means that one miRNA may regulate one or multiple target genes. Meanwhile, an individual gene may be regulated by multiple miRNAs [66]. Owing to the significant role of miRNAs in the intricate regulatory network of genes, there has been a surge in publications exploring their novel functions. For instance, Ghini *et al*. discovered a sophisticated regulatory layer involving specific endogenous targets that mediate miRNA gene expression in mammalian cells [67].

Indeed, the intervention or regulation mechanisms of miRNAs and genes in pathological processes are highly complex. These intricate miRNA regulatory details are documented in various databases. Researchers can select the appropriate database according to their needs to access relevant miRNA-gene regulatory relationships. A rigorously validated miRNA-gene interaction database by biological experiments, like miRTarbase [68] and TarBase [69], is always a trustworthy choice. Conversely, the databases that predict miRNA-gene target sites based on classical or machine learning algorithms, including TargetScan [70], miRDB [71] and targetMiner [72], offer insights into unknown miRNA-regulation network.

#### ceRNA network

In general, a part of non-coding RNAs, such as lncRNAs and circular RNAs (circRNAs), regulate gene expression by competitively binding to proteins or miRNAs, resulting in activation or inhibition [73]. This type of interaction, where molecules compete for binding to shared miRNAs, is referred to as a competing endogenous RNA (ceRNA) network. By sequestering miRNAs, ceRNAs can indirectly regulate the expression of target genes that are also regulated by those miRNAs. At the molecular level, a complex interplay among different RNA transcripts influences gene regulation in various biological processes [74]. Compared to miRNA-gene networks, ceRNA networks are more intricate and complex, involving a larger number of RNA molecules. As shown in Fig. 2**D**, the nodes within the ceRNA network encompass various types of RNA, including protein-coding mRNAs and non-coding RNAs such as miRNAs, lncRNAs, pseudogenic RNAs and circRNAs [75].

The ceRNA interaction network plays a crucial role in elucidating gene functions and regulatory mechanisms, and several identified ceRNA networks have been implicated in human diseases. For example, Sumazin *et al*. investigated the ceRNA activity of protein-coding mRNAs in a study on glioblastoma. They identified an extensive network of sponge interactions, mediating crosstalk between different regulatory pathways [76]. As the potential of the ceRNA network continues to be explored, the number of ceRNA interaction databases has been increasing. Prominent examples include LncACTdb [77] and DIANA-LncBase [78], which are centered around lncRNAs, starBase [79], miRSponge [80] and miRcode [81], which concentrate on miRNAs, ceRDB [82] and lnCeDB [83], specialized in the construction and analysis of competing ceRNA networks.

#### Protein-protein interaction (PPI) network

Protein-protein interactions form the basis of signaling pathways and bionetworks involved in various physiological processes [84], making PPI network construction a valuable approach for understanding cellular functions [85] as well as disease mechanisms [86], aiding drug design and repurposing [87], and deciphering subcellular gene interactions [88], [89] (as shown in Fig. 2**E**). To date, many important discoveries have been made through the use of PPI networks. For example, Fernández-Torras *et al*. demonstrated that gene modules within a PPI network can predict drug response, while Escala-Garcia *et al*. identified mediators of germline-driven variation in breast cancer prognosis through PPI network analysis [90], [91].

The databases containing PPI information serve as a cornerstone when building corresponding networks. The most prominent database in this regard is STRING, which specializes in constructing PPI networks based on the collected and rigorously assessed information of protein–protein interactions [49]. Another one is the IMEx consortium database, which aggregates protein interaction data from various high-throughput techniques and low-throughput experiments [92]. In addition, databases such as HuRI [93] and HPRD [94] focus on human interactions, while BioGRID [95] and InWeb [96] integrate data from multiple species. In addition to the typical databases mentioned above regarding PPI information, state-of-art artificial intelligence techniques have contributed to PPI networks by predicting the complex interactions between proteins, such as AlphaFold [97], Robetta (where RoseTTAFold is deployed) [98], DeepPPI [99], DeepConv-DTI [100] and DPPI [101]. Among these, AlphaFold is the most popular, achieving near-atomic accuracy in protein structure prediction and providing promising opportunities to uncover functional insights into the mechanisms of biological processes.

### Module analysis

Molecular networks are known to exhibit a high degree of modularity, with individual modules often consisting of genes (or proteins) that participate in the same biological functions. Modules can enhance functional genome annotation through the principle of guilt-by-association and contribute to a better understanding of disease pathogenesis and progression. As a result, module identification is often a critical step in extracting biological insights from network data. Up to date, a wide range of methods has been designed to recognize the communities (*i.e.*, modules) in bionetworks, including random walk-based methods, modularity optimization methods, local methods, kernel clustering methods, ensemble clustering methods, and hybrid methods [102], [103]. In terms of functionality, computationally predicted modules can be divided into three types, containing basic, co-expression and co-regulation modules. Basic modules represent a fundamental characteristic of many biological networks and are determined through high-density or correlation clustering. These modules share similar functions but not necessarily common expression or regulation patterns (Fig. 2**E-G**). In contrast, co-expression modules consist of genes with similar expression patterns that collectively serve a specific function, while co-regulation modules consist of genes that are regulated together by a shared regulatory program, influencing their behavior or participating in common biological processes.

#### Basic module analysis

An important property of the module is its ability to function independently of other modules, with its members having more connections to each other than to members of other modules, as reflected in the network topology [104]. The property of modular independence allows the application of various algorithms for analyzing the modules within networks (Fig. 2**E**). When tackling module identification, two prevalent approaches are the Infomap [105] and the MCODE [106]. Of which, Infomap is typically suitable for large-scale networks, exhibiting good robustness in effectively partitioning networks and identifying modules. However, it can be sensitive to parameters and entail longer computation time. Conversely, MCODE algorithm focuses on discovering locally dense subgraphs, making it effective for identifying functionally related substructures. Nevertheless, it may overlook global network structures and face higher computational complexity when dealing with large networks. Additionally, the Louvain community detection [107], based on modularity optimization, is renowned for efficiently identifying community structures in large networks. However, its performance can be influenced by the initial node selection, and it may not perform well in networks with overlapping communities. Collectively, the selection of algorithms should be based on careful consideration of the specific application scenarios and objectives, weighing their respective advantages and disadvantages.

#### Gene co-expression analysis

Divergent from algorithm-based module identification, co-expression refers to the phenomenon in which a set of genes share the same or relatively similar expression pattern concurrently (Fig. 2**F**). This phenomenon plays a pivotal role in shaping biological phenotypes, as the co-expression of genes is governed by intricate and integrated regulatory pathways spanning across multiple molecular levels [108]. In the pursuit of unraveling comprehensive co-expression networks from complex molecular alterations, researchers employ various methodologies. Among these, constructing co-expression networks based on weighted co-expression and clustering are prevalent. For instance, methods such as WGCNA [109], GeCON [110] and Petal [111] model the co-expression relationships between genes as weighted networks, where the weights represent the expression correlation between genes. In contrast to WGCNA's intricate thresholding, Petal identifies a co-expression network using an automatically defined threshold to indicate similar expression patterns. Additionally, CEMiTool facilitates automatic parameter selection and function enrichment analysis of modules, with more optimized parameter configurations than WGCNA and Petal [112]. Using these methodologies, researchers can systematically analyze gene co-expression networks, identify potential gene modules, and delve into their roles in biological processes, thereby providing vital clues for uncovering novel gene functionalities.

#### Gene co-regulated analysis

The genes that are simultaneously controlled or influenced by common regulators (*e.g.*, transcription factors, enhancers or repressors) are known as co-regulated genes, and they often occupy close positions on chromosomes, displaying associations within the same expression module or signaling pathway (Fig. 2**G**). Co-regulated genes share similar expression patterns due to shared regulators, which distinguishes them from co-expressed genes, which achieve similar expression patterns through different mechanisms. It is important to note that both co-regulation and independent co-expression may pass statistical tests designed to measure the similarity of expression patterns and/or the tightness of gene clusters relative to other clusters. Therefore, tools that focus solely on detecting clusters of similarly expressed genes may not effectively discriminate co-regulation from co-expression. For the construction of co-regulated modules, NetworkAnalyst and CoMoFinder [113] can be utilized. NetworkAnalyst is an online platform that supports integrative analysis of gene expression data through statistical, visual and regulated network approaches. CoMoFinder strives to discover reliable composite network motifs in co-regulatory networks consisting of miRNAs, TFs and genes.

Whenever possible, the use of diverse networks is recommended when conducting gene co-regulation analysis, as they contain complementary types of modules [103]. After community detection, over-representation analysis can be applied to unveil the functions of individual gene modules. Additionally, the association between module activity and observed phenotypes can be further investigated and clarified using GSEA.

### Knowledge Graph/text mining

**Fig. 3:**
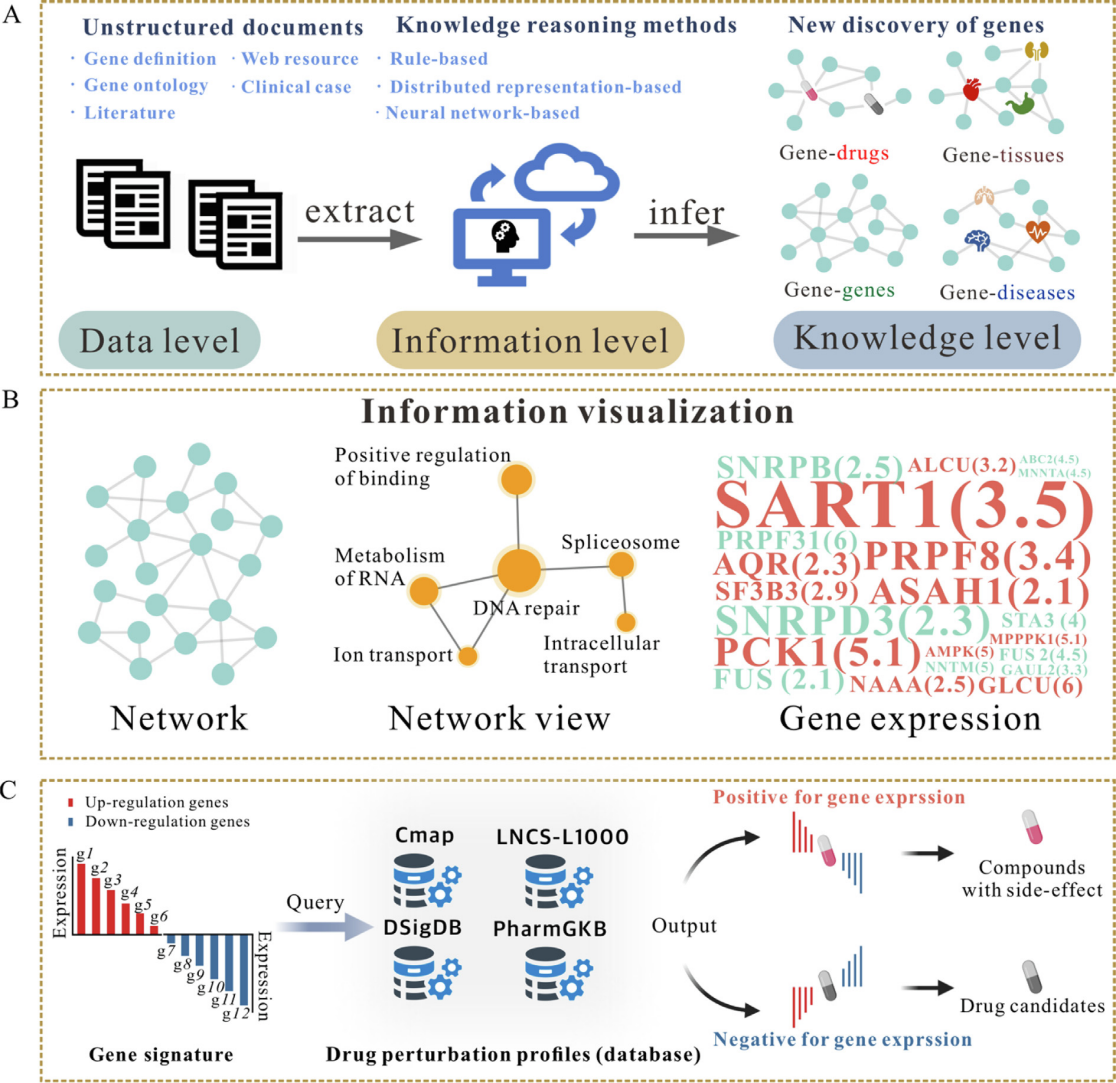
The analysis and visualization of KGs and potential drug discovery. **A)** The networks about genes extracted from literature. **B)** The nodes in graph represent data entities, and the edges represent the relationships between them. The network view depicts original nodes as enriched terms, with node size reflecting the weight of each term. Red nodes indicate up-regulation, green nodes indicate down-regulation, and numbers represent fold changes of DEGs. The size of the letters represents the significance of the P value. **C)** Gene signature-driven potential drug discovery. Initially, a query signature is prepared by compiling upregulated and downregulated genes associated with a disease state. This signature is then compared to a database of gene expression signatures from known perturbations or disease phenotypes. Compounds that exhibit a similar expression pattern to the disease state (inducing red and suppressing blue) are considered potential side-effect compounds. Conversely, compounds capable of reversing the disease expression pattern (suppressing red and inducing blue) are identified as candidate drugs.

Knowledge Graph (KG) has been proposed to discover new associations, patterns and knowledge by performing tasks such as information extraction, attribute definition, and creation of classification summaries against known data and relationships (Fig. 3**A**) [114]. KGs enhance the comprehensiveness and scientific insight of existing biological network data, enabling the prediction of novel associations between biological factors (such as gene signatures) and phenotypes [115]. This is particularly useful in domains like drug repurposing [116] and tumor research [115]. Currently, there are many popular tools for building KGs, and they are summarized in Supplementary Material Table S3.

#### Information extraction

With the rapid growth of scientific literature, manually locating and extracting relevant information is becoming increasingly challenging. Automated text extraction systems have emerged as a more efficient and comprehensive solution. In biomedicine, text mining often focuses on entity–entity interactions/relationships, such as drug-drug interactions (DDIs), PPIs/GRNs, protein-residue associations, or biological processes (such as phosphorylation). Notable software for text mining biomedical literature includes PubTator Central [117] and BEST [118]. PubTator Central specializes in automated annotation and tagging of texts from the PubMed database, while BEST helps users quickly locate information about specific entities within biomedical literature from multiple data sources.

#### Information assertions

The proliferation of biological data has created significant challenges in integrating and connecting related information from disparate sources. KG and text mining techniques can extract functional relationships and infer new relationships from massive amounts of literature. Additional information in KG can be automatically inferred through graph algorithms and logical reasoning. Knowledge reasoning methods are divided into three main categories: rule-based reasoning, distributed representation-based reasoning, and neural network-based reasoning [119]. Comprehensive application tools such as CROssBAR [120] and BioGraph [121] combine multiple technologies to extract, analyze, and visualize information from large amounts of literature. Another comprehensive tool, Phenolyzer [122], focuses on predicting potential genetic diseases and gene mutations based on genetic variation information and clinical phenotype data.

#### Information visualization

Word cloud is a popular information visualization method for quickly displaying terms with different frequencies (Fig. 3**B**), and has several applications in biomedicine [123], [124], [125], including the visualization of GO terms, visualization of pathway analysis results, analysis of literature and text mining, and clustering and annotation visualization. For instance, directly visualizing enrichment terms by their weights helps highlight the main biological processes associated with a group of genes, while filtering out redundant information. Similarly, key gene characteristics, including up- and down-regulation, fold changes between experimental and control groups, and P values, can be displayed using word clouds. Tools like WordCloud [126] and Gephi (https://gephi.org/) are widely used for generating word cloud and creating complex network graphs with various layout and customization options.

### Prediction of potential drugs

With the rise of high-throughput experimental techniques and the accumulation of omics data, transcriptome-based methods have become highly promising for drug repurposing [127], [128]. The fundamental idea of drug repurposing is that a specific drug induces unique gene expression signatures in cells, and comparing these gene expression signatures can establish connections with drug- or disease-induced phenotypes (Fig. 3**C**), thereby uncovering novel indications for existing drugs [129], [130].

Multiple large-scale databases have been developed based on this principle. One of the most famous is Connectivity Map (CMap), which consists of 6,100 gene expression profiles generated by exposing 1,309 compounds to five different cell lines at varying doses [131]. Since its inception, CMap has been instrumental in drug repurposing for cancers [132], neurological diseases [133], cardiovascular diseases [90], and other conditions. Currently, CMap has evolved into CMap2, also known as the LINCS-L1000 program [134], which encompasses 591,697 profiles derived from 29,668 compounds and genetic modifications (referred to as “perturbagens”) across 98 diverse cell lines. The substantial expansion in scale and breadth of CMap2 offers promising opportunities for enhanced pharmacogenomics investigations [133], [135].

In addition to the CMap series of databases, the Drug Signatures Database (DSigDB) is a widely utilized repository for drug gene-expression signatures [136]. DSigDB contains over 22,000 gene sets from different drugs, which can be used for drug-repurposing analysis using approaches such as signature similarity-based methods. Furthermore, PharmGKB is the most comprehensive pharmacogenomics knowledgebase, collecting extensive genotype and phenotype information linked to the pharmacogenome [137]. It contains data on 100 clinical dosing guidelines, 498 drug labels, 3,753 clinical annotations, 130 pathways, 65 pharmacogenes, and over 20,000 genetic variations.

Bioactive compounds from natural products in traditional Chinese medicine (TCM) represent a diverse and valuable source of potential drugs. To effectively identify candidate small molecules and active compounds that interact with the therapeutic targets and treat disease, a general strategy has been established, supported by various versatile and useful database resources. The process of predicting potential drug candidates typically involves integrating network pharmacology with multi-omics data, such as transcriptomics, proteomics, and metabolomics. The discovery of potentially effective components and active compounds from herb medicine can be divided into four main steps. The initial step is to decipher the chemical components within the TCM using text mining, database searching, and metabolomics technologies such as liquid chromatography/mass spectrometry (LC/MS), gas chromatography/mass spectrometry (GC/MS) and nuclear magnetic resonance (NMR) spectroscopy. Next, these components are screened against databases such as Traditional Chinese Medicine Systems Pharmacology Database (TCMSP) [138], based on the criteria like oral bioavailability, drug half-life and drug-likeness. In the third step, potential therapeutic targets for the disease or the chemical components are gathered or predicted through online databases. These include the Therapeutic Target Database (TTD) [139], TCMSP [138], SwissTargetPrediction [140], GeneCards [141], DrugBank [142] and PharmMapper [143], all of which provide target information related to diseases and chemical compounds. Once potential treatment target genes are obtained, a PPI network can be constructed, providing insights into interactions between chemical molecules and the targets. Finally, active compounds can be evaluated according to the compound-target interaction network using criteria such as the contribution index [144], ingredient efficacy scores [145], enrichment scoring algorithm based on a binomial statistical model [146] and maximal clique centrality algorithm [147].

In this process, transcriptomics data can also contribute to the discovery of potentially active small molecules and compounds with medicinal value. By revealing differential gene expression between control and diseased or treated samples, the statistically significant DEGs identified from transcriptomic analyses, particularly genes that are upregulated or uniquely expressed in patient tissues, may become potential therapeutic targets in future clinical trials [148], [149]. Apart from those predicted by algorithms and retrieved from databases, these potential target genes can also be used to construct compound-target networks and perform enrichment analysis. This helps prioritize hub genes, uncover functional categories, and provide new research perspectives. In summary, by integrating DEGs with network pharmacology, researchers can gain deeper insights into the biological processes affected by diseases or treatments and identify both therapeutic targets and potential drug candidates.

A summary of computational drug prediction tools is listed in Table 3, with additional tools provided in Supplementary Material Table S4.

**Table 3:** A summary of computational drug prediction tools.

**Name**	**Linking to DB**	**Resource**	**Year of release**	**PMID**
Clue	CMap, LINCS-L1000	https://clue.io/command	2017	29195078
Integrity	Integrity	https://integrity.clarivate.com/	2013	23593264
CREEDS	LINCS-L1000	https://amp.pharm.mssm.edu/CREEDS/	2016	27667448
L1000CDS^2^	LINCS-L1000	https://maayanlab.cloud/L1000CDS2/	2016	28413689
DvD	CMap, DrugBank, MeSH	Bioconductor package and Cytoscape plugin	2013	23129297
DeSigN	GDSC	http://design.cancerresearch.my/	2017	28198666
cogena	CMap, LINCS-L1000, CTD	Bioconductor package	2016	27234029
ksRepo	CTD	https://github.com/adam-sam-brown/ksRepo	2016	26860211
gene2drug	CMap	https://gene2drug.tigem.it/	2018	29236977
GeneExpressionSignature	CMap	Bioconductor package	2013	23374109
PDOD	CTD, DrugBank, MeSH	http://gto.kaist.ac.kr/pdod/index.php/main	2016	26818006
DTX	KEGG DRUG, DrugBank, NDB Open Data, PMDA JADER, Database relations	https://harrier.nagahama-i-bio.ac.jp/dtx/	2021	38097606
Phosprof	Reactome, PDB	https://phosprof.medals.jp/	2022	35994309

### Condition-specific gene expression analysis

#### Inferring spatial and temporal-specific gene expression patterns or markers

**Fig. 4:**
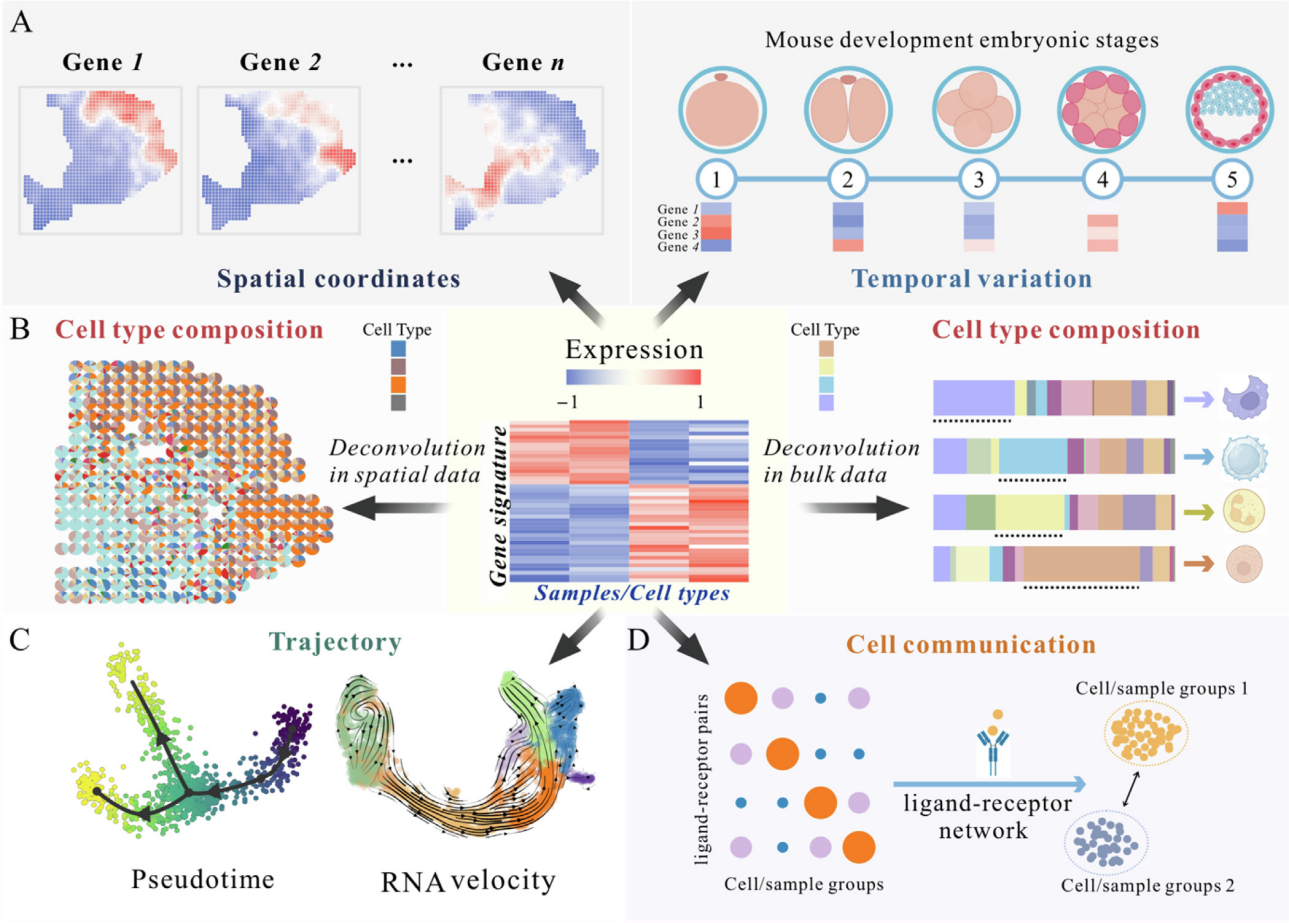
Overview of condition-specific analysis based on gene signatures. Gene signature with the ability to serve as the marker with **A)** spatial or temporal specificity, and usage for conducting **B)** cell deconvolution in spatial and bulk data, **C)** inferring trajectory, and **D)** uncovering cell–cell communication.

Although different tissues or developmental stages in organisms may share some common biological processes, their gene expression patterns vary significantly. This variation suggests that different regulatory mechanisms control spatial and temporal specificity [150]. Understanding the specific expression and regulation of genes in these contexts is essential for exploring genetic relationships, the etiology of tissues and developmental stages, and discovering new therapeutic targets (Fig. 4**A**). For example, *SIRT1* regulates glucose and fatty acid metabolism in the liver but inhibits fat mobilization in adipose tissues during fasting [151]. Additionally, *SIRT1* expression is high at certain stages of mouse embryonic development but declines with further organogenesis [152]. Such variations underscore the distinct regulatory mechanisms that shape gene expression patterns across different spatial and temporal domains.

In research practice, several large-scale bio-projects and initiatives (*e.g.*, GTEx [153], Expression Atlas [154], Human Proteome Map [155], and RNA-Seq Atlas [156]) provide valuable data on gene expression levels and patterns across various tissues and developmental time points (Additional software and tools are listed in Table 4). All these resources enhance our understanding of the spatial and temporal dynamics of gene expression within organisms.

**Table 4:** Several query tools for tissue and developmental stage-specific gene expression.

**Name**	**Statistical method**	**Resource**	**Year of release**	**PMID**
GEPIA	TPM cut-off	http://gepia.cancer-pku.cn/	2017	28407145
HumanBase	Bayesian integration	https://hb.flatironinstitute.org	2015	25915600
Expression Atlas	FPKM cut-off	https://www.ebi.ac.uk/gxa	2010	19906730
ToppCluster	Hypergeometric test	https://toppcluster.cchmc.org	2010	20484371
TISSUES	confidence score	http://tissues.jensenlab.org and Cytoscape plugin	2015	26157623
TissueEnrich	Hypergeometric test	https://tissueenrich.gdcb.iastate.edu/	2019	30346488
ORGANizer	Hypergeometric test	geneorganizer.huji.ac.il	2017	28444223
deTS	Fisher’s exact test, *t*-test	CRAN package	2019	30824912
TS-GOEA	Hypergeometric test	https://bioinformaticshome.com/tools/rna-seq/descriptions/TS-GOEA.html	2019	31760951
TEnGExA	FPKM cut-off	http://webtom.cabgrid.res.in/tissue_enrich/ and github package	2021	32960209
Dynamic-BM	FPKM cut-off	http://bioinfo.ibp.ac.cn/Dynamic-BM/	2018	28575155
ADEIP	Two tailed Mann Whitney *U* test	http://gb.whu.edu.cn/ADEIP/	2021	34254996
IID	Expression cut-off	https://ophid.utoronto.ca/iid	2016	26516188
GENT	ANOVA or *t*-test	http://gent2.appex.kr/gent2/	2019	31296229
diseaseQUEST	Wilcoxon rank sum test	https://github.com/FunctionLab/diseasequest-docker/	2018	30346941
WebCSEA	Permutation-based cell-type specificity test	https://bioinfo.uth.edu/webcsea/	2022	35610053

#### Discovering tissue-specific gene markers (or cell markers)

When analyzing bulk RNA-seq data, DEGs that are upregulated in specific tissues are typically defined as tissue-specific gene markers. After further thorough experimental validation, these markers can be used for tissue labeling, targeted treatment of disease, and organ development studies. Similarly, in scRNA-seq, numerous cell type-specific DEGs (particularly those that are upregulated) have been identified and recommended as cell markers through experimental studies and scRNA-seq analysis, aiding in cell annotation [157], [158]. The identification of cell markers has been carefully performed and validated in many publications, and relevant databases have been gradually developed, *e.g.*, CellMarker [159] and PlantCellMarker [160]. These cell (or tissue) marker genes, discovered through additional analysis of DEGs, have found wide applications in various fields, including cell and tissue identification, analysis of complex tissue microenvironment, pseudotime analysis of cells, RNA stability analysis, and prediction of cell communication. In this review, we focus on the significance of cell type-specific markers derived from DEGs and their potential biological implications.

(1) Estimation of cell type composition in complex tissue

As shown in Fig. 4**B**, cell markers can be used for estimating cell type compositions and proportions within complex tissues [161]. There are two primary types of technologies that utilize gene signatures along with expression profile data to deduce the cellular composition of mixed samples: enrichment-based methods and deconvolution algorithms [162]. The former approach typically requires assessing the enrichment scores of individual cell types, where cell type-specific genes are highly expressed in the sample of interest and expressed at lower levels in other samples [163]. Prominent examples of enrichment methods include MCPcounter [164], xCell [162], ImmuCellAI [165]. However, it is important to note that incomplete or inaccurate gene lists can lead to incorrect estimates of cell type enrichment. Moreover, genes specific to certain cell types may also be expressed in other cell types, introducing errors in enrichment analysis. By mathematically modeling mixed data, deconvolution algorithms can estimate the contribution of each cell type in mixed samples, which helps overcome the challenges in estimating cell type enrichment. Deconvolution algorithms enable quantitative estimation of cell type proportions by utilizing cell type-specific gene expression signatures [166], dividing into three main classes: linear regression approach [167], integer linear programming approach [168], and machine learning approach [169]. These deconvolution methods have been developed with an emphasis on various perspectives. For instance, CIBERSORT [169] and TIMER2.0 [170] are tailored for the identification and quantification of immune cell types, MuSiC [14] primarily addresses sample heterogeneity and technical variability issues in single-cell data, while EPIC [171] is predominantly utilized for identifying and analyzing DNA methylation patterns within individual cells. The more cell composition analysis tools are summarized in Supplementary Material Table S5**.**

(2) Dynamics inference: pseudotime and RNA velocity analysis

In a single-cell transcriptome analysis, trajectory inference seeks to predict the evolving patterns in a single-cell transcriptome landscape by considering each cell's transcriptome as a fixed snapshot at a specific time point within a cellular process. These sequential snapshots form a dynamic trajectory illustrating cells' progression through varying states, commonly referred to as a “pseudo-temporal trajectory” [172]. For trajectory inference, the computational burden is a major constraint. To minimize computational consumption, the highly variable features usually are used to carry out trajectory inference (Fig. 4**C**). In actual practice, DEGs are often treated as highly variable genes for cell trajectory inference and subsequent analysis.

Currently, there are two main strategies for cell trajectory analysis, including pseudotime analysis [173] and RNA velocity analysis [174]. Pseudotime analysis allows us to reconstruct dynamic biological processes without sampling tissues at different time points, identify critical transition points between distinct cell states, and analyze shifts in cell-type composition and cell synchronization. RNA velocity is a computational technique that estimates the future transcriptional trajectory of individual cells by analyzing the relative abundances of spliced and unspliced mRNA, leveraging the assumption that unspliced mRNA represents nascent transcripts while spliced mRNA reflects mature, stable transcripts [175].

In the past few years, several popular trajectory inference tools have been released one after another, such as Monocle [173], Slingshot [176], TIMEOR [177], scVelo [178], CellRank [179], and VeloViz [180]. As a prevail trajectory inference method, Monocle3 adopts an enhanced principal graph-embedding procedure to refine the details of learned trajectories, reduce the running time and enable the identification of loop-structured cellular development. Besides, it also has unique versatility, featured by the additional functions of identifying genes with trajectory-dependent expression and allowing users to visualize the analytical results in different ways. In pseudotime analysis, Slingshot primarily focuses on pseudotime inference and transition point identification [176], while TIMEOR is more dedicated to recognizing time information and conducting time-series analysis [177]. To overcome the limitations of the original RNA velocity model [175], scVelo employs a likelihood-based dynamical model, which effectively infers gene-specific transcriptional dynamics and resolves distinct kinetics in heterogeneous subpopulations. CellRank facilitates pseudotime and RNA velocity analyses, especially for handling branching and cyclic cellular developmental pathways [179]. VeloViz is an R package that provides the rich visualization capabilities and customization options [180]. More detailed information on these tools can be found in Supplementary Material Table S6.

(3) Cell-cell communication prediction

Cell communication across multiple cell types and tissues extensively relies on interactions between secreted ligands (such as hormones, growth factors, chemokines, cytokines, and neurotransmitters) and cell-surface receptors [181], [182], and plays a critical role in the regulation of early embryonic development, tissue and organ development, tumorigenesis, and cross-cellular metabolic homeostasis [183], [184], [185], [186]. Typically, signaling events between cells are mediated by protein interactions, such as ligand-receptor binding (Fig. 4**D**). Transcriptome data (*e.g.*, DNA microarray, bulk RNA-seq and scRNA-seq data) are commonly recommended for analyzing cell communication due to their accessibility compared to proteomics. These data is applied to infer cellular communication by predicting ligand-receptor interactions based on the differential expression of ligands and receptors between different cell types or samples [181].

To enhance cellular communication analysis, databases of ligand-receptor pairs and corresponding computational tools are continually evolving. These resources provide essential support for investigating intercellular communication. Notably, databases and tools vary in their focus and scope. CellPhoneDB [187] specializes in intercellular signaling, particularly ligand-receptor interactions, while ConnectomeDB [188] focuses on brain connectomics, analyzing connectivity patterns within the brain. Several computational tools have been developed to calculate communication scores between different cells or samples according to gene expression profiles. While a considerable portion of these tools is primarily tailored for single-cell transcriptomic data, CellChat [189], CellCall [189], iTALK [190], SpaOTsc [191] and scTensor [192]), are capable of analyzing intercellular communication in bulk transcriptomic data. Among these, SpaOTsc integrates spatial and transcriptomic information, enabling the analysis of spatial distribution and communication patterns between cells. For additional details on these tools and databases, refer to Supplementary Material Table S7**.**

### Sample label prediction

**Fig. 5:**
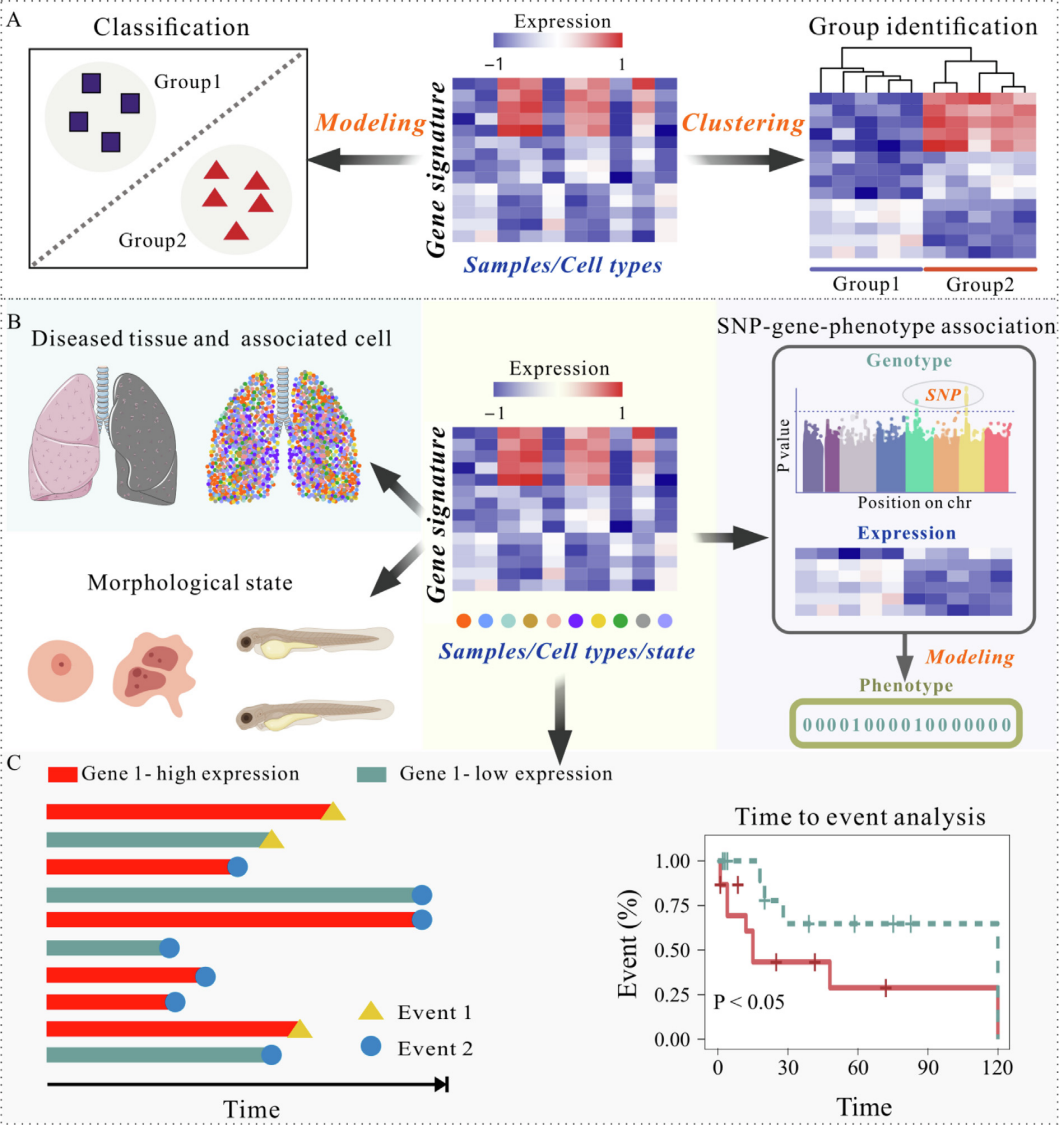
Illustration of sample label prediction and gene-phenotype association analysis. **A)** The sample label prediction mainly relies on two strategies, classification prediction and clustering analysis. **B)** Gene and phenotype association analysis. Based on gene expression level, the interactions between genes and phenotypes (*e.g.*, disease, tissue, cell state, cell or organism morphology) were inferred based on machine learning algorithms. The single-cell genomics provides a means to quantitatively annotate cell states on the basis of high-information content and high-throughput measurements according to the gene expression level. The SNP-gene-phenotype association strategy consists of two kinds: (1) Direct model development. This model is based on SNP-gene-phenotype association using data integration algorithms with gene clusters (sets) and expression levels. SNP clusters (sets) corresponding to the selected gene clusters can be identified by eQTL data. (2) Modeling using reference panel: the TWAS strategy. TWAS consists of three steps: (i) Modeling based on a reference panel to establish the relationship between SNPs and gene expression levels. Samples in the reference panel have genotype and expression level data for fitting the relationship between these SNP loci and corresponding gene expression levels (selecting SNP loci within 500 kb or 1 M range upstream and downstream of the gene). (ii) Using the model in step (1) to predict the gene expression levels for another set of individuals with genotype data. (iii) Analyzing the association between genes and phenotype using predicted gene expression levels. **C)** The principle of time-to-event (survival) analysis.

The prediction of sample labels is a critical challenge in scenarios where there is a need to discover new groups or enhance diagnostic accuracy with limited labeled samples. Classification and clustering methods are two types of well-established and effective machine learning approaches to address this problem. As shown in Fig. 5**A**, classification methods can predict the categories of samples or the types of cells based on gene lists, making them widely applicable in fields such as single-cell genomics and spatial transcriptome analyses. On the other hand, clustering analysis divides data into subsets, grouping similar patterns together based on DEGs [193]. This approach is also valuable for exploring subgroups within a dataset and uncovering new functions of genes within the same cluster. Additionally, clustering analysis can also be used to establish relationships between subgroups and clinical annotations or to assess batch effects in samples. A number of clustering tools offering a variety of algorithms and the ability to visualize the analysis results are listed in Table 5.

**Table 5:** The popular classification and clustering tools based on gene expression profiles.

**Name**	**Description**	**Resource**	**Remark**
**Supervised learning: classification model development tools**			
caret	·streamline the process of model training for classification.	https://topepo.github.io/caret/index.html	https://doi.org/10.18637/jss.v028.i05
Tidymodels	·building models using tidyverse principles.	https://www.tidymodels.org/packages/	−
mlr3verse	·data.table and R6 ·parallel computing ·building “graph” flow learners ·unified interface ·advanced machine learning algorithms	https://github.com/mlr-org/mlr3	https://doi.org/10.21105/joss.01903
MASS	·providing multiple datasets ·basic models and statistical algorithms	https://cran.r-project.org/web/packages/MASS/index.html	https://www.stats.ox.ac.uk/pub/MASS4/
**Unsupervised learning: clustering tools**			
Nbclust	·30 indexes for determining the optimal number of clusters ·providing the best clustering scheme	https://cran.r-project.org/web/packages/NbClust/	https://doi.org/10.18637/jss.v061.i06
ClustVis	·user-friendly and clustering visualization web tool	https://biit.cs.ut.ee/clustvis/	PMID: 25969447
TimeClust	·user-friendly software package to cluster genes according to temporal expression profiles. ·two original algorithms expressed designed for clustering short time series together ·Windows and LINUX platforms can be downloaded free	http://aimed11.unipv.it/TimeClust/	PMID: 18065427
Medusa	·highly interactive and it supports weighted and multi-edged graphs ·a variety of layout and clustering methods for visualization	https://sites.google.com/site/medusa3visualization	PMID: 21978489
wcd	·compact memory-large files can be clustered on a single processor and very fast	https://code.google.com/p/wcdest	PMID: 18480101
cola	·helps users to select optimal parameter values. ·provides rich functionalities to apply multiple partitioning methods in parallel and directly compare their results ·generates a comprehensive HTML report.	Bioconductor package	PMID: 33275159
hiplot	·open and advanced one-stop biomedical visualization and analysis platform with various modules	https://hiplot.cn/	PMID: 35788820

### Gene-phenotype association analysis

#### Transcriptome-wide association study (TWAS)

Gene-environment interactions are fundamental in determining individual traits, or phenotypes, which include molecular or cellular characteristics, morphological traits, behaviours, and so on (Fig. 5**B**). To delve into the specific molecular mechanisms underlying the relationship between gene expression quantitative loci (eQTL) and phenotypes, transcriptome-wide association studies (TWAS), akin to GWAS [194], have emerged as an important new strategy. TWAS uses genotype data to estimate gene expression based on reference transcriptomic datasets (*e.g.*, GTEx) and then links the predicted gene expression with phenotypic traits to identify gene-trait associations. By correlating these estimated expression phenotypes with disease phenotypes, researchers can identify gene expression changes associated with diseases. This approach reveals critical gene expression changes relevant to disease prediction, diagnosis, and treatment. TWAS is particularly useful for identifying functional genes regulated by disease-associated variants, thereby providing insights into disease mechanisms and other phenotypic characteristics [195]. Recently, single-cell genomics has gained increasing traction as an effective approach to overcome this limitation, and this technology has demonstrated superior capability in investigating the correlation between gene expression levels and eQTL strength across diverse cell types [196].

Over the past decade, several methodologies have been developed for conducting TWAS and analyzing the associations between single-nucleotide polymorphisms (SNPs)/genes and phenotypes to uncover genetic variations linked to complex human diseases or traits. These findings have unveiled new connections between genes and traits, enhancing our comprehension of the complexities in various traits and finding practical applications across diverse clinical settings [197], [198]. Prominent tools in TWAS analysis include FUSION [199], PrediXcan [200], MetaXcan [201], SMR [202], and so on. Of which, FUSION [199] integrates various genotype and expression data, providing a robust statistical framework for association analysis. PrediXcan [200] and MetaXcan [201], employ linear regression models to estimate gene expression and correlate it with disease phenotypes, supporting a range of phenotypic combinations and offering enhanced statistical capabilities and visualization tools. SMR identifies significant associations between the expression levels of certain genes and complex traits using summary data from GWAS and eQTL studies [202]. These tools are crucial for TWAS analysis and are widely used in various fields, such as medicine and agriculture. For example, Li *et al.* conducted a systematic analysis of gene expression, structural variations and alternative splicing in soybeans using SMR and FUSION, to investigate the genetic basis of traits at the gene level [203]. You *et al.* used FUSION to determine the association between gene expression and fiber quality traits in their study on the regulatory controls of duplicated gene expression during fiber development in allotetraploid cotton [204].

#### Time-to-event analysis

Time-to-event analysis, also called survival analysis, encompasses a learning framework and a range of techniques employed to estimate the duration until specific events occur based on observed data (Fig. 5**C**). In biomedicine, time-to-event analysis is widely used to evaluate the influence of gene expression levels on different events (*e.g.*, patient progress, disease recurrence, biological persistence, and animal behavior [205]). Valuable findings can be obtained by constructing a curve based on gene expression deviations. For instance, an upregulated gene expression may indicate a shorter time until death, or the presence of an upregulated gene post-treatment could predict a favorable prognosis.

To handle events that are not binary or variables that change over time, multistate modeling has been explored [206]. While the Kaplan-Meier and Cox regression methods are commonly used statistical techniques in time-to-event analysis, the field has progressively shifted towards incorporating different machine learning methods, such as random forest, Naïve Bayes, and K-nearest neighbors.

## DEGMiner, a comprehensive website hub was developed

**Fig. 6:**
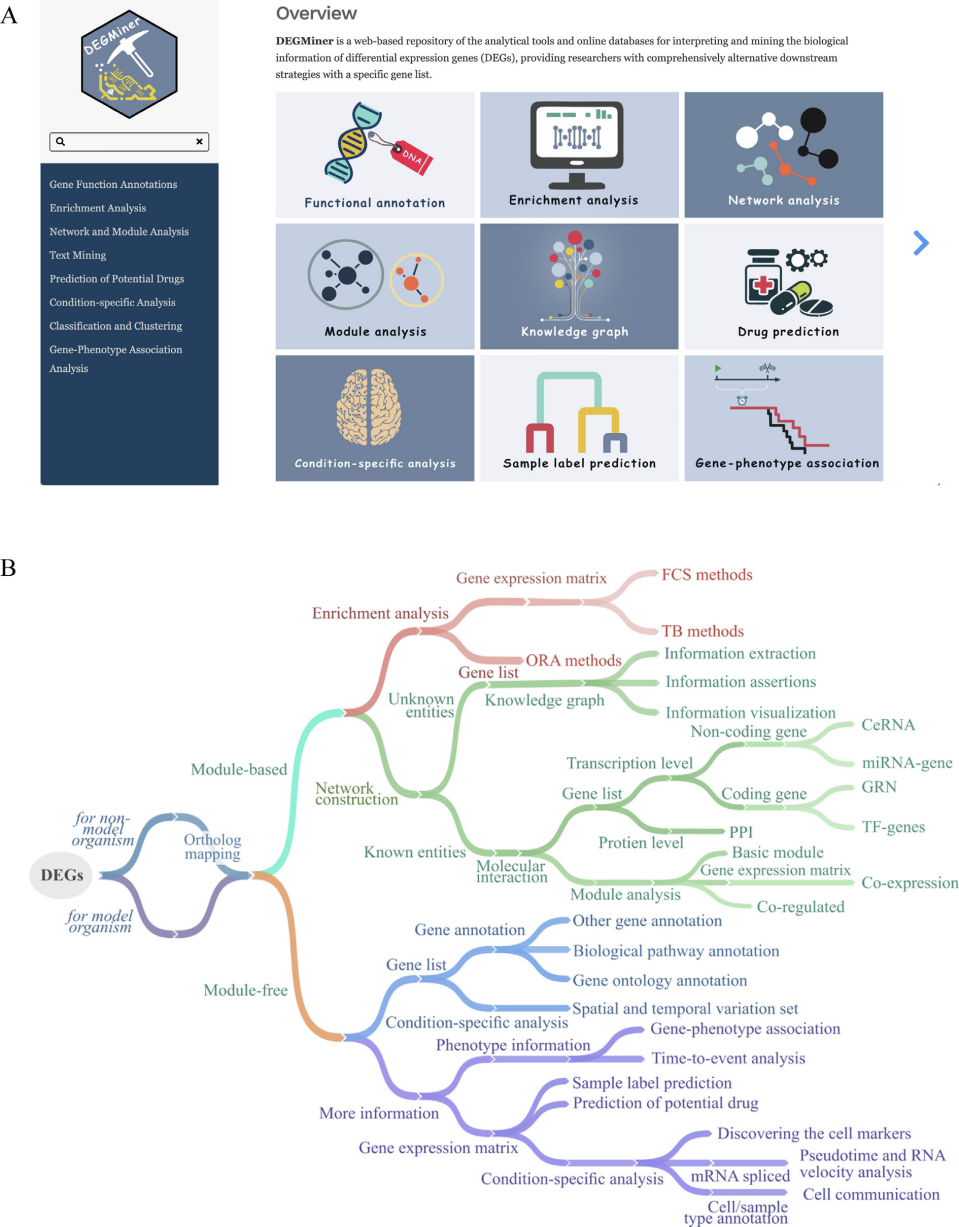
DEGMiner website and practical guidelines for users. **A)** The homepage of DEGMiner website (https://www.ciblab.net/DEGminer/). **B)** Practical guidelines for users.

Given the lack of a comprehensive guide to the analysis of DEGs and their subsequent implications, we have created a robust online platform called DEGMiner (https://www.ciblab.net/DEGMiner/). This platform, developed using shiny and rmarkdown under R (Fig. 6**A**), deploys a centralized repository of analytical tools and web-based databases that facilitate the interpretation and exploration of biological data associated with DEGs. DEGMiner offers researchers a comprehensive selection of alternative downstream strategies for a specific gene list. With over 300 tools and databases covering nine kinds of strategies for DEG analysis, this website serves as a valuable resource. Additionally, DEGMiner collects features and additional information about the databases, as well as installation instructions, environmental deployment details, and other metadata to provide users with a preliminary understanding. The primary goal of DEGMiner is to support readers in quickly and easily finding tools for analyzing DEGs, thereby reducing the time spent on manual searching. It lays a solid foundation for numerous bioinformatics studies and helps beginners understand the molecular mechanisms underlying the DEGs.

## Risks and challenges in practice

Due to insufficient attention in certain fields, excessive dependence on prior information, intrinsic limitation of analytical approaches, and usage preferences, the downstream analysis of gene lists poses four primary challenges, which we mainly focus on for further discussion and elucidation.

(1) The annotation and enrichment of non-model species have not received adequate attention, and many tools lack support for these species. In bioinformatics analysis, it is essential to facilitate the convenient and rapid conversion of homologous genes from non-model species to model species. Therefore, the development of robust and user-friendly homologous gene conversion tools is an urgent priority.
(2) Network analysis usually depends on proven knowledge bases. Since there is little prior knowledge about gene-small molecule interactions, it is difficult to infer such interactive relationships, construct the network and track the upstream or downstream regulatory molecules of target genes. In such cases, we suggest searching for conserved motifs among sequences through BLAST [207], and subsequently targeting potential elements that regulate gene activity within commonly conserved regions in the absence of the specifically curated and summarized interaction information.
(3) Computational drug repurposing approach based on gene expression changes closely links gene expression to drug treatment. It saves time and money, as well as reduces the possibility of identifying drugs with high toxicity to some extent. However, pharmacologically relevant effects may not be primarily reflected at the transcriptional level. Moreover, the current database consists of a small number of compounds (compared to the large number of drugs currently available), and different treatment durations can also lead to batch effects in the results [208].
(4) When it comes to extensively exploring the information related to DEGs, a major future trend is the creation of comprehensive tools that are more user-friendly, efficient, and accessible, without the need for a coding background. Alternatively, some complex methods (with numerous parameter settings) will be designed to be “simpler”, such as built-in deep learning capabilities, which can directly recommend suitable parameters or optimal results based on the characteristics of the data. In many cases, a tool or method providing a number of parameters may create a “complexity trap” and it will be easily abandoned by users if it takes a long time to learn the excessive parameters before using them.

Overall, the development trend for tools should be towards a simple operation that mobilizes complex operations and then obtains the optimal results.

## Discussion and suggestion

DEGs are commonly used to characterize genetic differences between two or more biological sample groups, in support of specific hypothesis-driven studies [209], [210]. However, analyzing a large number of DEGs poses challenges and requires careful consideration when applying bioinformatics methods. Therefore, it is essential to have a systematic and directional analysis workflow.

In this review, we not only present a complete methodology for the analysis of DEGs but also offer a practical guide for researchers to choose the appropriate methods (Fig. 6**B**). First, users should consider whether converting genes into orthologs of model species is necessary to ensure broader applicability in various analysis processes. If researchers are specifically interested in gene interactions, they may opt for module-based methods; otherwise, they can choose module-free methods. Gene function enrichment should be prioritized if the focus is on gene function or roles in particular pathways. For detailed gene interactions, network construction is recommended, and two types of topologies are possible. Known entities from different networks may offer meaningful insights, while unknown entities from knowledge graphs may lead to new discoveries. Transcription or protein-level regulatory interactions can be chosen if the focus is on known coding genes, with miRNA regulation network and ceRNA for regulated interactions of non-coding genes and GRN and TF regulation for regulated interactions of coding genes. If the researchers are interested in module or community interactions of genes, the module analysis would be suitable for downstream projects, followed by additional network analysis.

If researchers focus on a few genes and related molecular mechanisms, the module-free methods should be utilized (Fig. 6**B**). Gene annotation and condition-specific analysis are effective ways to obtain functional information about genes. Particularly, gene annotation can provide basic functional descriptions when the enrichment service does not return significant (*i.e.*, P value > 0.05) enriched terms, or when there are few genes in the analysis. Researchers can also use other relevant knowledge to mine biological information, such as phenotype information about time and state for survival analysis, SNP data and gene expression data for gene-phenotype association analysis, gene expression matrix for drug repurposing, cluster analysis, cell marker discovery, trajectory analysis, and cell communication. In addition, RNA velocity analysis can be performed with the help of mRNA spliced information, and cell/sample annotation can contribute to cell communication inference.

## Conclusion and prospects

To better understand the identified DEGs, various methods and tools have been developed to place these findings within a broader biological context [211]. In this review, we systematically summarized and discussed the strategies for conducting a comprehensive analysis of DEGs, aiming to maximize the biological insights from transcriptome data. The DEG analysis methods discussed here can also be applied to proteomics data to a great extent. This suggests the possibility of integrating various omics datasets, starting from genes, to comprehensively understand biological processes. Specifically, we outline nine strategies for mining biological information from DEGs, with detailed descriptions of relevant tools in their respective sections. We also highlight the advantages and limitations of these methods. For reader convenience, we provide an online resource that consolidates all the mentioned databases or tools. This practical guide, along with the summarized tools and data available on the website, serves as a valuable reference for researchers without a strong background in bioinformatics.

Despite existing methodological and technological challenges, numerous opportunities are available to enhance our understanding of life activities by exploring the wealth of data information at hand. In future research examining the biological importance and relevance of DEGs using bioinformatics and computational biology approaches, it will be crucial to take into account the following key considerations: 1) Integrated omics profiling: Integrating diverse omics data types (*e.g.*, genomics, transcriptomics, proteomics) provides a more comprehensive view of DEGs and their functional implications. Developing algorithms and tools for multi-omics data integration is crucial for gaining insights into complex biological processes. 2) Gene-phenotype association: Large cohorts are essential for human phenome studies, but limitations arise when exploring gene-phenotype connections. Future tools should address challenges posed by small sample sizes. 3) Machine learning and predictive modeling: Leveraging machine learning algorithms and predictive models helps identify patterns in DEG datasets, predict gene functions, and infer regulatory relationships. Customized machine learning methods are crucial for analyzing the complexity of biological data effectively. 4) Dimensionality reduction techniques: Techniques like principal component analysis or t-distributed stochastic neighbor embedding aid in visualizing and interpreting high-dimensional DEG datasets. These methods help identify key features and reduce noise in complex gene expression data, particularly useful for single-cell data analysis.

**Ethics Statement**.

*No clinical trials and animal experiments were performed in this study.*

## CRediT authorship contribution statement

**Huachun Yin:** Investigation, Writing – original draft, Writing – review & editing, Visualization. **Hongrui Duo:** Web Construction, Writing – review & editing. **Song Li:** Supervision, Funding acquisition. **Dan Qin:** Writing – review & editing. Lingling Xie: Writing – review & editing. **Yingxue Xiao:** Writing – review & editing. **Jing Sun:** Writing – review & editing. **Jingxin Tao:** Web Construction. **Xiaoxi Zhang:** Writing – review & editing. **Yinghong Li:** Resources, Visualization. **Yue Zou:** Writing – review & editing, Data collection. **Qingxia Yang:** Web Construction. **Xian Yang:** Resources, Supervision. **Youjin Hao:** Writing – review & editing, Supervision, Funding acquisition. **Bo Li:** Conceptualization, Supervision, Funding acquisition, Project administration, Writing – review & editing.

## Funding

This work was sponsored by Natural Science Foundation of Chongqing, China (No. CSTC2019JCYJ-MSXMX0527), Science and Technology Research Program of Chongqing Municipal Education Commission (No. KJQN202100538, KJQN202100642), 10.13039/501100001809National Natural Science Foundation of China (62101087), 10.13039/501100002858China Postdoctoral Science Foundation (2021MD703942), and Open Fund of Yunnan Key Laboratory of Plant Reproductive Adaptation and Evolutionary Ecology, Yunnan University (YNPRAEC-2023004) .

## Declaration of competing interest

The authors declare that they have no known competing financial interests or personal relationships that could have appeared to influence the work reported in this paper.
