# Supplementary material for: Unlocking biological insights from differentially expressed genes: Concepts, methods, and future perspectives
Source: J Adv Res. 2024 Dec 6;76:135–57. doi: 10.1016/j.jare.2024.12.004 (PMC12793742; doi:10.1016/j.jare.2024.12.004)
Supplement: Supplementary Data 1 [file mmc1.docx]

**Supplementary Material for:**

**Unlocking Biological Insights from Differentially Expressed Genes: Concepts, Methods, and Future Perspectives**

Huachun Yin^1,2,3,†^, Hongrui Duo^1,†^, Song Li^2^, Dan Qin^4^, Lingling Xie^1^, Yingxue Xiao^1^, Jing Sun^1^, Jingxin Tao^1^, Xiaoxi Zhang^1^, Yinghong Li^5^, Yue Zou^1^, Qingxia Yang^6^, Xian Yang^1^, Youjin Hao^1,*^ and Bo Li^1,*^

1 College of Life Sciences, Chongqing Normal University, Chongqing 401331, China;

2 Department of Neurosurgery, Xinqiao Hospital, The Army Medical University, Chongqing 400037, China;

3 Department of Neurobiology, Chongqing Key Laboratory of Neurobiology, The Army Medical University, Chongqing 400038, China;

4 Department of Biology, College of Science, Northeastern University, Boston, MA 02115, USA;

5 Chongqing Key Laboratory of Big Data for Bio Intelligence, Chongqing University of Posts and Telecommunications, Chongqing 400065, P. R. China;

6 Zhejiang Provincial Key Laboratory of Precision Diagnosis and Therapy for Major Gynecological Diseases, Women's Hospital, Zhejiang University School of Medicine, Hangzhou 310058, P. R. China.

***** Corresponding author.

E-mail addresses: libcell@cqnu.edu.cn (B. Li), haoyoujin@hotmail.com (Y.J. Hao).

† These authors contributed equally to this work.

This article contains Supplementary Material, Supplementary Table S1–S7

**Table S1 The commonly used bioinformatics tools for enrichment analysis**

| **Name** | **Key statistical method** | **Resource** | |
| --- | --- | --- | --- |
| **Synthesize enrichment tools (GO, pathway and more terms)** | | |  |
| DAVID[^1^](#_ENREF_1) | Fisher's exact test (modified as EASE score) | https://david.ncifcrf.gov | |
| GSEA[^2^](#_ENREF_2) | Kolmogorov-Smirnov-like test | https://www.gsea-msigdb.org | |
| clusterProfiler[^3^](#_ENREF_3) | Hypergeometric test | Bioconductor package | |
| Enrichr[^4^](#_ENREF_4) | Fisher’s exact test | https://maayanlab.cloud/Enrichr/ | |
| PANTHER[^5^](#_ENREF_5) | Binomial test, Fisher's exact test | http://pantherdb.org | |
| ClueGO[^6^](#_ENREF_6) | Hypergeometric test | Cytoscape plugin | |
| IPA[^7^](#_ENREF_7) | Right-tailed Fisher Exact test | http://www.ingenuity.com | |
| KOBAS[^8^](#_ENREF_8) | Binomial test, chi-square test, Fisher's exact test, hypergeometric test | http://kobas.cbi.pku.edu.cn/kobas3 | |
| Toppgene[^9^](#_ENREF_9) | Fisher’s inverse chi-square, hypergeometric test | http://toppgene.cchmc.org | |
| EnrichmentMap[^10^](#_ENREF_10) | Fisher’s exact test | Cytoscape plugin | |
| Metascape[^11^](#_ENREF_11) | Hypergeometric test | http://metascape.org/ | |
| WebGestalt[^12^](#_ENREF_12) | Hypergeometric test | http://www.webgestalt.org/option.php | |
| STEM[^13^](#_ENREF_13) | Binomial test, Hypergeometric test | http://sb.cs.cmu.edu/stem/ | |
| g:Profiler[^14^](#_ENREF_14) | Hypergeometric test | <http://biit.cs.ut.ee/gprofiler/> and CRAN package | |
| GAGE[^15^](#_ENREF_15) | Meta-test | Bioconductor package | |
| PAGE[^16^](#_ENREF_16) | Z-score | Python | |
| GeneCodis[^17^](#_ENREF_17) | Hypergeometric test, chi-square test | https://genecodis.genyo.es/ | |
| iDEP | student’s t-test | http://ge-lab.org/idep/ | |
| GeneTrail[^18^](#_ENREF_18) | Hypergeometric test, Kolmogorov-Smirnov test | http://genetrail.bioinf.uni-sb.de | |
| Babelomics[^19^](#_ENREF_19) | Fisher's Exact test, T-test | http://www.babelomics.org | |
| PageMan[^20^](#_ENREF_20) | Fisher's exact test, Chi-square test, Wilcoxon test | https://mapman.gabipd.org/mapman | |
| ErmineJ[^21^](#_ENREF_21) | Permutations test, Wilcoxon test, rank-sum test | https://erminej.msl.ubc.ca | |
| EnrichNet[^22^](#_ENREF_22) | Xd-distance | http://www.enrichnet.org/ | |
| DOSE[^23^](#_ENREF_23) | Hypergeometric test | Bioconductor package | |
| L2L[^24^](#_ENREF_24) | Binomial test, Hypergeometric test | <http://depts.washington.edu/l2l/> (Not available) | |
| SciMiner[^25^](#_ENREF_25) | Fisher’s exact test | http://hurlab.med.und.edu/SciMiner/ | |
| CoPub[^26^](#_ENREF_26) | Fisher’s exact test | http://services.nbic.nl/cgi-bin/copub/CoPub.pl | |
| FunNet[^27^](#_ENREF_27) | Fisher’s exact test | http://www.funnet.info | |
| ArrayXPath[^28^](#_ENREF_28) | Fisher’s exact test | http://www.snubi.org/software/ArrayXPath/ | |
| ProfCom[^29^](#_ENREF_29) | Greedy heuristics | http://webclu.bio.wzw.tum.de/profcom/ | |
| CLEAN[^30^](#_ENREF_30) | Hypergeometric test, Fisher’s exact test | http://eh3.uc.edu/clean/ | |
| FIDEA[^31^](#_ENREF_31) | Hypergeometric test | http://www.biocomputing.it/fidea | |
| N2C[^32^](#_ENREF_32) | T-test, Z-score | http://www.maayanlab.net/N2C | |
| HPOSim[^33^](#_ENREF_33) | Hypergeometric test | CRAN package | |
| PAEA[^34^](#_ENREF_34) | χ2 test, mean p value test, median p value test, Wilcoxon rank sum test, the weighed Kolmogorov-Smirnov test | <http://amp.pharm.mssm.edu/PAEA> (Not available) | |
| hypeR[^35^](#_ENREF_35) | Hypergeometric test | https://github.com/montilab/hypeR | |
| CIE[^36^](#_ENREF_36) | Fisher’s exact test, Ternary and Quaternary scoring statistic | https://umbibio.math.umb.edu/cie/app | |
| GeneAnswers[^37^](#_ENREF_37) | Hypergeometric test | Bioconductor package | |
| SIGNAL | Z scores | https://signal.niaid.nih.gov | |
| **GO enrichment tools** | |  | |
| BiNGO[^38^](#_ENREF_38) | Hypergeometric test, Binomial test | Cytoscape plugin | |
| GOrilla | Hypergeometric test | http://cbl-gorilla.cs.technion.ac.il | |
| WEGO[^39^](#_ENREF_39) | Chi-square test | http://wego.genomics.cn | |
| agriGO[^40^](#_ENREF_40) | Hypergeometric test, Fisher's exact test | http://systemsbiology.cau.edu.cn/agriGOv2/ | |
| GOstats[^41^](#_ENREF_41) | Hypergeometric test | Bioconductor package | |
| GOTermFinder[^42^](#_ENREF_42) | Hypergeometric test | https://go.princeton.edu/cgi-bin/GOTermFinder | |
| GoMiner[^43^](#_ENREF_43) | Fisher’s exact test | https://discover.nci.nih.gov/gominer/index.jsp | |
| GOStat[^44^](#_ENREF_44) | Kolmogorov-Smirnov statistics | http://gostat.wehi.edu.au | |
| topGO[^45^](#_ENREF_45) | Kolmogorov-Smirnov test, Fisher’s exact test, Goeman's globaltest, t-test | Bioconductor package | |
| GOEAST[^46^](#_ENREF_46) | Hypergeometric test | http://omicslab.genetics.ac.cn/GOEAST/ | |
| FuncAssociate[^47^](#_ENREF_47) | Fisher’s exact test | http://llama.mshri.on.ca/funcassociate_client/html/ | |
| Onto-Express[^48^](#_ENREF_48) | Fisher's exact test, hypergeometric test, Binomial test, Chi-square test | http://vortex.cs.wayne.edu:8080 | |
| Ontologizer | Fisher’s exact test | http://ontologizer.de/webontologizer/ | |
| GOToolBox | Fisher's exact test, Binomial test, Permutations test, Hypergeometric test | https://www.webcitation.org/ | |
| GOSSIP[^49^](#_ENREF_49) | Fisher’s exact test | http://gossip.gene-groups.net | |
| GOSim[^50^](#_ENREF_50) | Resnik's similarity | CRAN package | |
| GOHyperGAll[^41^](#_ENREF_41) | Hypergeometric test | Bioconductor package GOstats | |
| EasyGO[^51^](#_ENREF_51) | Hypergeometric test, Chi-square test, Binomial test | http://bioinformatics.cau.edu.cn/easygo/ | |
| GoSurfer[^52^](#_ENREF_52) | Chi-square test | http://systemsbio.ucsd.edu/GoSurfer/ | |
| GeneTools[^53^](#_ENREF_53) | Fisher’s exact test | http://www.genetools.no | |
| ShinyGO[^54^](#_ENREF_54) | Chi-square test, T-test | http://bioinformatics.sdstate.edu/go/ | |
| GOLEM[^55^](#_ENREF_55) | Hypergeometric test | http://function.princeton.edu/GOLEM | |
| GOFFA[^56^](#_ENREF_56) | Fisher's inverse chi-Squared test | http://edkb.fda.gov/webstart/arraytrack/ | |
| BayGO[^57^](#_ENREF_57) | Bayesian, Goodman and Kruskal's gamma factor | http://blasto.iq.usp.br/~tkoide/BayGO | |
| OntologyTraverser | Hypergeometric test, Fisher’s exact test | CRAN package | |
| ADGO[^58^](#_ENREF_58) | Z-statistic, Fisher's exact test, Hypergeometric test | http://www.btool.org/ADGO2 | |
| JProGO[^59^](#_ENREF_59) | Kolmogorov-Smirnov test, t-test, Wilcoxon's test | http://www.jprogo.de | |
| Gobar[^60^](#_ENREF_60) | Hypergeometric test | http://katahdin.cshl.org:9331/GO | |
| GOCluster[^61^](#_ENREF_61) | Hypergeometric test | Bioconductor package | |
| FuncCluster[^62^](#_ENREF_62) | Fisher's exact test | CRAN package | |
| Tmod | Fisher's exact test, hypergeometric test, U test, CERNO test | CRAN package | |
| MamPhEA[^63^](#_ENREF_63) | Fisher's exact test | http://evol.nhri.org.tw/MamPhEA/ | |
| ProbCD[^64^](#_ENREF_64) | Yule's Q test, Goodman-Kruskal's gamma test, Cramer's test | http://xerad.systemsbiology.net/ProbCD/ | |
| netGO[^65^](#_ENREF_65) | Fisher’s exact test | https://github.com/unistbig/netGO | |
| Blast2GO[^66^](#_ENREF_66) | Gossip, Fisher’s exact test | http://www.blast2go.de | |
| **Pathway enrichment tools** | |  | |
| SPEED2 | Bates test or Chi-square test | https://speed2.sys-bio.net/index.html | |
| TACCO | hypergeometric test | http://tacco.life.nctu.edu.tw/ | |
| PathVisio | Hypergeometric test, Z-scores | http://www.pathvisio.org | |
| NetWalker[^67^](#_ENREF_67) | T-test | http://netwalkersuite.org | |
| Graphite Web[^68^](#_ENREF_68) | Hypergeometric test, Fisher’s exact test, Global Test | https://graphiteweb.bio.unipd.it/ | |
| FunSet[^69^](#_ENREF_69) | Hypergeometric test | http://funset.uno | |
| PaintOmics | Fisher’s Exact test and Stouffer’s method | https://www.paintomics.org/ | |
| HisCoM-PAGE | Machine learning | R code: http://statgen.snu.ac.kr/software/HisCom-PAGE/Download.zip | |
| PAGWAS | Fisher's exact test or Chi-square test | CRAN package | |
| DecoPath | Fisher's exact test or GSEA Pre-Ranked | <https://decopath.scai.fraunhofer.de/> and https://github.com/DecoPath/DecoPath | |
| CPA | ORA, Wilcox test, Kolmogorov–Smirnov test, GSA, GSEA, FGSEA, PADOG | http://cpa.tinnguyen-lab.com | |

**Table S2 The tools and databases used for network analysis and visualization**

| **Database** | | | | | | | | | |
| --- | --- | --- | --- | --- | --- | --- | --- | --- | --- |
| **Type** | | | **Name** | **Resource** | | | | | |
| Protein-Protein | | | STRING[^70^](#_ENREF_70) | https://string-db.org | | | | |  |
|  |  |  | HPRD[^71^](#_ENREF_71) | http://www.hprd.org/ (not available) | | | | |  |
|  |  |  | IntAct[^72^](#_ENREF_72) | https://www.ebi.ac.uk/intact/home | | | | |  |
|  |  |  | BIND[^73^](#_ENREF_73) | http://bond.unleashedinformatics.com/Action? (not available) | | | | |  |
|  |  |  | DIP[^74^](#_ENREF_74) | https://dip.doe-mbi.ucla.edu/dip/Main.cgi | | | | |  |
|  |  |  | BioGRID[^75^](#_ENREF_75) | https://thebiogrid.org/ | | | | |  |
|  |  |  | MINT[^76^](#_ENREF_76) | https://mint.bio.uniroma2.it/ | | | | |  |
|  |  |  | InnateDB[^77^](#_ENREF_77) | https://www.innatedb.com/index.jsp | | | | |  |
|  |  |  | HuRI[^78^](#_ENREF_78) | http://www.interactome-atlas.org/ | | | | |  |
|  |  |  | IMEx[^79^](#_ENREF_79) | http://www.imexconsortium.org/ | | | | |  |
|  |  |  | InWeb[^80^](#_ENREF_80) | http://www.inweb.gr/ | | | | |  |
|  |  |  | NDEx[^81^](#_ENREF_81) | https://www.ndexbio.org/#/ | | | | |  |
|  |  |  | IID[^82^](#_ENREF_82) | http://iid.ophid.utoronto.ca/ | | | | |  |
|  |  |  | SIGNOR[^83^](#_ENREF_83) | https://signor.uniroma2.it/ | | | | |  |
|  |  |  | MatrixDB[^84^](#_ENREF_84) | http://matrixdb.univ-lyon1.fr/ | | | | |  |
|  |  |  | I2D[^85^](#_ENREF_85) | http://ophid.utoronto.ca/ophidv2.204/index.jsp | | | | |  |
|  |  |  | SPIKE[^86^](#_ENREF_86) | https://www.cs.tau.ac.il/~spike/ | | | | |  |
| TF-gene | | | JASPAR[^87^](#_ENREF_87) | https://jaspar.genereg.net/ | | | | |  |
|  |  |  | CHEA[^88^](#_ENREF_88) | http://amp.pharm.mssm.edu/lib/chea.jsp | | | | |  |
|  |  |  | AnimalTFDB[^89^](#_ENREF_89) | http://bioinfo.life.hust.edu.cn/AnimalTFDB4/#/ | | | | |  |
|  |  |  | TRED[^90^](#_ENREF_90) | http://rulai.cshl.edu/ (not available) | | | | |  |
|  |  |  | RegNetwork[^91^](#_ENREF_91) | https://regnetworkweb.org/ | | | | |  |
|  | | | PlantCARE[^92^](#_ENREF_92) | https://bioinformatics.psb.ugent.be/webtools/plantcare/html/ | | | | |  |
| miRNA-gene | | | TargetScan[^93^](#_ENREF_93) | https://www.targetscan.org/vert_80/ | | | | |  |
|  |  |  | miRBase[^94^](#_ENREF_94) | https://www.mirbase.org/ | | | | |  |
|  |  |  | miRDB[^95^](#_ENREF_95) | http://www.mirdb.org/ | | | | |  |
|  |  |  | miRecords[^96^](#_ENREF_96) | http://miRecords.umn.edu/miRecords (not available) | | | | |  |
|  |  |  | RNAhybrid[^97^](#_ENREF_97) | https://bibiserv.cebitec.uni-bielefeld.de/rnahybrid | | | | |  |
|  |  |  | miRTarbase[^98^](#_ENREF_98) | https://mirtarbase.cuhk.edu.cn/~miRTarBase/miRTarBase_2022/php/index.php | | | | |  |
|  |  |  | miR2Disease[^99^](#_ENREF_99) | http://www.mir2disease.org/ | | | | |  |
|  |  |  | TarBase[^100^](#_ENREF_100) | https://dianalab.e-ce.uth.gr/html/diana/web/index.php?r=tarbasev8 | | | | |  |
|  |  |  | mirDIP[^101^](#_ENREF_101) | http://ophid.utoronto.ca/mirDIP/ | | | | |  |
|  |  |  | targetMiner[^102^](#_ENREF_102) | www.isical.ac.in/∼bioinfo_miu (not available) | | | | |  |
| ceRNA | Comprehensive | | ENCODE[^103^](#_ENREF_103) | https://www.encodeproject.org/ | | | | |  |
|  | RNA Interactomes | | starbase[^104^](#_ENREF_104) | https://starbase.sysu.edu.cn/index.php | | | | |  |
|  |  |  | MiRcode[^105^](#_ENREF_105) | http://www.mircode.org/ | | | | |  |
|  |  |  | DIANA-LncBase[^106^](#_ENREF_106) | https://diana.e-ce.uth.gr/lncbasev3 | | | | |  |
|  |  |  | OmniPath[^107^](#_ENREF_107) | https://omnipathdb.org/ | | | | |  |
|  |  |  | miRSponge[^108^](#_ENREF_108) | http://www.bio-bigdata.net/miRSponge (not available) | | | | |  |
|  |  |  | ceRDB[^109^](#_ENREF_109) | https://www.oncomir.umn.edu/cefinder/basic_search.php | | | | |  |
|  |  |  | LncACTdb[^110^](#_ENREF_110) | http://bio-bigdata.hrbmu.edu.cn/LncACTdb/ | | | | |  |
|  |  |  | PceRBase[^111^](#_ENREF_111) | http://bis.zju.edu.cn/pcernadb/index.jsp | | | | |  |
| **Tool** | | | | | | | | | |
| **Name** | | **Resource** | | | **gene-gene** | **gene→gene** | **TF→gene** | **miRNA→gene** | |
| Cytoscape | | https://cytoscape.org | | | ✓ | ✓ | ✓ | ✓ | |
| GeneMANIA[^112^](#_ENREF_112) | | <https://genemania.org> and Cytoscape plugin | | | ✓ | 🗶 | ✓ | ✓ | |
| NetworkAnalyst [^113^](#_ENREF_113) | | <https://www.networkanalyst.ca> and CRAN R package | | | ✓ | ✓ | 🗶 | 🗶 | |
| ConsensusPathDB[^114^](#_ENREF_114) | | http://cpdb.molgen.mpg.de | | | ✓ | ✓ | ✓ | ✓ | |
| [iRegulon](https://journals.plos.org/ploscompbiol/article?id=10.1371/journal.pcbi.1003731)[^115^](#_ENREF_115) | | Cytoscape plugin | | | ✓ | ✓ | ✓ | ✓ | |
| NAViGaTOR[^116^](#_ENREF_116) | | http://ophid.utoronto.ca/navigator/ | | | ✓ | ✓ | 🗶 | 🗶 | |
| BioProfiling.de[^117^](#_ENREF_117) | | http://www.bioprofiling.de/index.html (not available) | | | ✓ | 🗶 | 🗶 | ✓ | |
| FunCoup[^118^](#_ENREF_118) | | http://FunCoup.sbc.su.se | | | ✓ | 🗶 | ✓ | ✓ | |
| miRTargetLink[^119^](#_ENREF_119) | | https://ccb-web.cs.uni-saarland.de/mirtargetlink/ and https://ccb-compute.cs.uni-saarland.de/mirtargetlink2/ | | | 🗶 | 🗶 | 🗶 | ✓ | |
| SNOW[^120^](#_ENREF_120) | | http://www.babelomics.org (not available) | | | ✓ | 🗶 | 🗶 | 🗶 | |
| FFLtool[^121^](#_ENREF_121) | | http://bioinfo.life.hust.edu.cn/FFLtool/ | | | 🗶 | 🗶 | ✓ | ✓ | |

**Table S3 A summary of tools for constructing knowledge graph**

| **Name** | **Resource** |
| --- | --- |
| PubTator central[^122^](#_ENREF_122) | https://www.ncbi.nlm.nih.gov/research/pubtator/ |
| Phenolyzer[^123^](#_ENREF_123) | https://compbio.charite.de/phenomizer/ |
| BioGraph[^124^](#_ENREF_124) | https://biograph.be/ |
| WordCloud[^125^](#_ENREF_125) | https://apps.cytoscape.org/apps/wordcloud |
| BEST[^126^](#_ENREF_126) | http://best.korea.ac.kr/ |
| iPTMnet[^127^](#_ENREF_127) | http://proteininformationresource.org/iPTMnet |
| Genes2WordCloud[^128^](#_ENREF_128) | http://www.maayanlab.net/G2W |
| TeamTat[^129^](#_ENREF_129) | https://www.teamtat.org/ |
| CADA[^130^](#_ENREF_130) | https://cada.gene-talk.de/webservice |
| CROssBAR[^131^](#_ENREF_131) | https://crossbar.kansil.org/ |

**Table S4 A summary of tools for drug repurposing**

| **Name** | **Linking to DB** | **Resource** |
| --- | --- | --- |
| Clue[^132^](#_ENREF_132) | Cmap, LINCS | https://clue.io/command |
| Integrity[^133^](#_ENREF_133) | Integrity | http://integrity.thomson-pharma.com (Not valid) |
| CREEDS[^134^](#_ENREF_134) | LINCS | http://amp.pharm.mssm.edu/CREEDS/ |
| L1000CDS2[^135^](#_ENREF_135) | LINCS | https://maayanlab.cloud/L1000CDS2/#/index |
| PharmacoGx[^136^](#_ENREF_136) | GDSC and CCLE | Bioconductor package and https://github.com/bhklab/PharmacoGx |
| DrugComboRanker[^137^](#_ENREF_137) | Cmap | https://github.com/methodistsmab/DrugComboRanker |
| SEP-L1000[^138^](#_ENREF_138) | LINCS | http://maayanlab.net/SEP-L1000/ |
| L1000FWD[^139^](#_ENREF_139) | LINCS | https://maayanlab.cloud/L1000FWD/ |
| Mantra[^140^](#_ENREF_140) | GEPs | https://mantra.tigem.it/Default.aspx |
| DSEA[^141^](#_ENREF_141) | Cmap | http://dsea.tigem.it |
| DvD[^142^](#_ENREF_142) | Cmap, DrugBank, MeSH | Bioconductor package and https://saezlab.github.io/DrugVsDisease/ |
| cogena[^143^](#_ENREF_143) | Cmap, LINCS, CTD | Bioconductor package |
| gene2drug[^144^](#_ENREF_144) | Cmap | https://gene2drug.tigem.it/ |
| DeSigN[^145^](#_ENREF_145) | GDSC | http://design.cancerresearch.my/ |
| ksRepo[^146^](#_ENREF_146) | CTD | http://github.com/adam-sam-brown/ksRepo |
| iLINCS[^147^](#_ENREF_147) | Cmap and LINCS | http://www.ilincs.org/ilincs/ |
| GeneExpressionSignature[^148^](#_ENREF_148) | Cmap | https://github.com/yiluheihei/GeneExpressionSignature |
| PDOD[^149^](#_ENREF_149) | CTD, DrugBank, MeSH | http://gto.kaist.ac.kr/pdod/index.php/main |
| SigCom LINCS[^150^](#_ENREF_150) | LINCS, GTEx, and GEO | https://maayanlab.cloud/sigcom-lincs |
| DTX | KEGG DRUG,  DrugBank,  NDB Open Data,  PMDA JADER, | https://harrier.nagahama-i-bio.ac.jp/dtx/ |
| Phosprof | Reactome, PDB | https://phosprof.medals.jp/ |

GEPs: a compendium of gene expression profiles (GEPs) following drug treatment of human cell lines.

**Table S5 Available bioinformatics tools for cell composition analysis**

| **Name** | **Algorithm type** | **Resource** |
| --- | --- | --- |
| CIERSORT[^151^](#_ENREF_151) | ML | https://cibersort.stanford.edu/index.php |
| ESTIMATE[^152^](#_ENREF_152) | ssGSEA | <https://bioinformatics.mdanderson.org/estimate/> and CRAN package |
| xCell[^153^](#_ENREF_153) | ssGSEA | http://xCell.ucsf.edu/ and https://github.com/dviraran/xCell |
| TIMER2.0[^154^](#_ENREF_154) | ML, LS, ILP | https://cistrome.shinyapps.io/timer/ |
| MCPcounter[^155^](#_ENREF_155) | mean of marker gene expression | http://github.com/ebecht/MCPcounter |
| CIBERSORTx[^156^](#_ENREF_156) | ML | https://cibersortx.stanford.edu |
| Bseq_SC[^157^](#_ENREF_157) | ML | http://github.com/shenorrlab/bseq-sc |
| EPIC[^158^](#_ENREF_158) | LS | http://epic.gfellerlab.org/ and https://github.com/GfellerLab/EPIC |
| quanTIseq[^159^](#_ENREF_159) | LS | https://icbi.i-med.ac.at/software/quantiseq/doc/ |
| Abbas *et al.*[*^160^*](#_ENREF_160) | LS | https://doi.org/10.1371/journal.pone.0006098 |
| ABIS[^161^](#_ENREF_161) | ML | <https://giannimonaco.shinyapps.io/ABIS/> and  https://github.com/giannimonaco/ABIS |
| ImmuCellAI[^162^](#_ENREF_162) | ssGSEA | http://bioinfo.life.hust.edu.cn/ImmuCellAI#!/ |
| MuSiC[^163^](#_ENREF_163) | ML | https://github.com/xuranw/MuSiC |
| DeconRNASeq[^164^](#_ENREF_164) | QP | Bioconductor package |
| Max Schelker *et al.*[*^165^*](#_ENREF_165) | SVR | https://figshare.com/s/711d3fb2bd3288c8483a |
| DSA[^166^](#_ENREF_166) | QP | https://github.com/zhandong/DSA |
| singscore[^167^](#_ENREF_167) | Rank | Bioconductor package |
| Ting Gong *et al.*[*^168^*](#_ENREF_168) | QP | https://doi.org/10.1371/journal.pone.0027156 |
| PERT[^169^](#_ENREF_169) | LS | https://doi.org/10.1371/journal.pcbi.1002838.s003 |
| ImmuCC[^170^](#_ENREF_170) | v-SVR | https://github.com/wuaipinglab/ImmuCC |
| immunoStates[^171^](#_ENREF_171) | v-SVR | https://wiki.khatrilab.stanford.edu/immunoStates |
| DCQ[^172^](#_ENREF_172) | ML | http://dcq.tau.ac.il/ |
| SCDC[^173^](#_ENREF_173) | ENSEMBLE method | https://meichendong.github.io/SCDC/ |
| Edec[^174^](#_ENREF_174) | Non-negative matrix factorization | https://github.com/BRL-BCM/EDec |
| MMAD[^175^](#_ENREF_175) | ML | https://sourceforge.net/projects/mmad/ |
| scBio[^176^](#_ENREF_176) | SVR | CRAN package and https://github.com/amitfrish/scBio |
| debCAM[^177^](#_ENREF_177) | Convex analysis of mixtures and non-negative least squares | Bioconductor package |
| TEMT[^178^](#_ENREF_178) | Expectation-Maximization | https://github.com/uci-cbcl/TEMT |
| DeMixT[^179^](#_ENREF_179) | ML | https://github.com/wwylab/DeMixTallmaterials |
| Dtangle[^180^](#_ENREF_180) | Linear model | https://gjhunt.github.io/dtangle/ |
| FARDEEP[^181^](#_ENREF_181) | Least trimmed square | https://github.com/YuningHao/FARDEEP |
| TOAST[^182^](#_ENREF_182) | Iterated matrix factorization | Bioconductor package |
| CDSeq[^183^](#_ENREF_183) | ML | https://github.com/kkang7/CDSeq_R_Package |
| Casey P. Shannon *et al.*[*^184^*](#_ENREF_184) | QP | https://doi.org/10.1371/journal.pone.0095224 |
| deconvSeq[^185^](#_ENREF_185) | GM | https://github.com/rosedu1/deconvSeq/ |
| Xiaoqing Yu *et al.*[*^186^*](#_ENREF_186) | v-SVR | https://doi.org/10.1186/s12885-019-5927-3 |
| Digitaldlsorter[^187^](#_ENREF_187) | ML | https://github.com/cartof/digitalDLSorter |
| DeClust[^188^](#_ENREF_188) | ML | https://github.com/integrativenetworkbiology/DeClust |
| MySort[^189^](#_ENREF_189) | SVR | https://testtoolshed.g2.bx.psu.edu/view/moneycat/mysort/e3afe097e80a |
| smcgenedeconv[^190^](#_ENREF_190) | Sequential monte carlo | https://github.com/moyanre/smcgenedeconv |
| Chiu Yen-Jung *et al.*[*^191^*](#_ENREF_191) | v-SVR | https://github.com/holiday01/deconvolution-to-estimate-immune-cell-subsets |
| NITUMID[^192^](#_ENREF_192) | Non-negative matrix factorization | https://github.com/tdw1221/NITUMID |
| ADAPTS[^193^](#_ENREF_193) | Hierarchical | https://github.com/sdanzige/ADAPTS |
| MOMF[^194^](#_ENREF_194) | Non-negative matrix factorization | https://github.com/sqsun/MOMF |
| Deblender[^195^](#_ENREF_195) | semi/unsupervised multi-operational | https://github.com/kondim1983/Deblender/ |
| Lishuang Qi *et al.*[*^196^*](#_ENREF_196) | Non-negative matrix factorization | https://doi.org/10.1371/journal.pone.0100934 |
| MIXTURE[^197^](#_ENREF_197) | v-SVR | https://github.com/elmerfer/MIXTURE.App |
| Semi-CAM[^198^](#_ENREF_198) | Non-negative matrix factorization | https://github.com/ylidong/semi-CAM |
| DTD[^199^](#_ENREF_199) | Correlation | https://github.com/MarianSchoen/DTD |
| Infino[^200^](#_ENREF_200) | Bayesian | https://github.com/hammerlab/infino |

*ML: machine learning approach; *LS: least squares regression; *ILP: integer linear programming approach; QP: Quadratic programming; GM: Generalized linear model; v-SVR: v-support vector regression.

**Table S6 The bioinformatics tools for trajectory inference**

| **Name** | **Type** | **Resource** |
| --- | --- | --- |
| Monocle3[^201^](#_ENREF_201) | pseudotime | https://github.com/cole-trapnell-lab/monocle3 |
| Slingshot[^202^](#_ENREF_202) | pseudotime | https://github.com/kstreet13/slingshot |
| CytoTree | pseudotime | https://ytdai.github.io/CytoTree/index.html |
| CellRank[^203^](#_ENREF_203) | pseudotime and RNA velocity | https://cellrank.org/ |
| scVelo[^201^](#_ENREF_201) | pseudotime and RNA velocity | https://github.com/theislab/scvelo |
| VeloViz[^204^](#_ENREF_204) | RNA velocity | https://github.com/JEFworks-Lab/veloviz |
| Asc-Seurat | pseudotime | <https://asc-seurat.readthedocs.io/en/latest/index.html>, https://github.com/KirstLab/asc_seurat/ |
| VeTra[^205^](#_ENREF_205) | RNA velocity | https://github.com/wgzgithub/VeTra |
| TIMEOR[^206^](#_ENREF_206) | pseudotime | https://timeor.brown.edu/app/timeor |
| UniTVelo[^207^](#_ENREF_207) | RNA velocity | https://github.com/StatBiomed/UniTVelo |
| TIPS[^208^](#_ENREF_208) | pseudotime | https://github.com/qingshanni/TIPS |
| Velo-Predictor[^209^](#_ENREF_209) | RNA velocity | https://github.com/clay001/Velo-Predictor |
| ORIGINS[^210^](#_ENREF_210) | pseudotime | https://github.com/danielasenraoka/ORIGINS |

**Table S7 Existing ligand-receptor pair databases and tools for measuring cell-cell communication**

| **Name** | **Type** | **Resource** |
| --- | --- | --- |
| IUPHAR[^211^](#_ENREF_211) | Database | https://www.guidetopharmacology.org/ |
| CellPhoneDB[^212^](#_ENREF_212) | Database, repository | <https://www.cellphonedb.org/> and https://github.com/Teichlab/cellphonedb |
| FANTOM5[^213^](#_ENREF_213) | Database | https://fantom.gsc.riken.jp/5/suppl/Ramilowski_et_al_2015/ |
| CellCellInteractions[^214^](#_ENREF_214) | Database | http://baderlab.org/CellCellInteractions |
| ligand-receptor pairs in literature[^215^](#_ENREF_215) | Repository | https://github.com/LewisLabUCSD/Ligand-Receptor-Pairs |
| DLRP[^216^](#_ENREF_216) | Database | https://dip.doe-mbi.ucla.edu/dip/DLRP.cgi |
| ConnectomeDB[^217^](#_ENREF_217) | Database | https://db.humanconnectome.org |
| CellTalkDB[^218^](#_ENREF_218) | Database | http://tcm.zju.edu.cn/celltalkdb/ |
| ICELLNET[^219^](#_ENREF_219) | Repository | https://github.com/soumelis-lab/ICELLNET |
| Cellinker[^220^](#_ENREF_220) | Database | http://www.rna-society.org/cellinker/ |
| PlantPhoneDB[^221^](#_ENREF_221) | Database | https://jasonxu.shinyapps.io/PlantPhoneDB/ |
| cellcallEXT[^222^](#_ENREF_222) | Repository | https://github.com/shouguog/cellcallEXT |
| **Method** | **Name** | **Resource** |
| Nonparametric tests | CellPhoneDB | https://github.com/Teichlab/cellphonedb |
|  | CellChat[^223^](#_ENREF_223) | <http://www.cellchat.org/> and https://github.com/sqjin/CellChat |
|  | Giotto[^224^](#_ENREF_224) | https://github.com/RubD/Giotto_site |
|  | SingleCellSignalR[^225^](#_ENREF_225) | https://github.com/SCA-IRCM/SingleCellSignalR |
|  | ICELLNET | https://github.com/soumelis-lab/ICELLNET |
|  | CellCall[^226^](#_ENREF_226) | https://github.com/ShellyCoder/cellcall |
|  | TraSig[^227^](#_ENREF_227) | https://github.com/doraadong/TraSig |
|  | cellcallEXT | https://github.com/shouguog/cellcallEXT |
| Differential combinations | celltalker[^220^](#_ENREF_220) | https://arc85.github.io/celltalker/index.html |
|  | iTALK[^228^](#_ENREF_228) | https://github.com/Coolgenome/iTALK |
|  | PyMINEr[^229^](#_ENREF_229) | https://www. sciencescott.com/ pyminer |
|  | DeepCCI[^230^](#_ENREF_230) | https://github.com/JiangBioLab/DeepCCI |
| Graph or network | NicheNet[^231^](#_ENREF_231) | https://github.com/saeyslab/nichenetr |
|  | SpaOTsc[^232^](#_ENREF_232) | https://github.com/zcang/SpaOTsc |
|  | CCCExplorer[^233^](#_ENREF_233) | https://github.com/methodistsmab/CCCExplorer |
|  | SoptSC[^234^](#_ENREF_234) | https://mkarikom.github.io/RSoptSC/index.html |
|  | NATMI[^235^](#_ENREF_235) | https://github.com/forrest-lab/NATMI/ |
|  | scConnect[^236^](#_ENREF_236) | https://github.com/JonETJakobsson/scConnect |
|  | FunRes[^237^](#_ENREF_237) | https://git-r3lab.uni.lu/kartikeya.singh/funres |
| Tensor based | scTensor[^238^](#_ENREF_238) | https://github.com/rikenbit/scTensor |

**REFERENCES:**

1. Huang da, W., Sherman, B.T. & Lempicki, R.A. Systematic and integrative analysis of large gene lists using DAVID bioinformatics resources. *Nat Protoc* **4**, 44-57 (2009).

2. Subramanian, A.*, et al.* Gene set enrichment analysis: a knowledge-based approach for interpreting genome-wide expression profiles. *Proc Natl Acad Sci USA* **102**, 15545-15550 (2005).

3. Yu, G., Wang, L.G., Han, Y. & He, Q.Y. clusterProfiler: an R package for comparing biological themes among gene clusters. *OMICS* **16**, 284-287 (2012).

4. Kuleshov, M.V.*, et al.* Enrichr: a comprehensive gene set enrichment analysis web server 2016 update. *Nucleic Acids Res* **44**, W90-97 (2016).

5. Thomas, P.D.*, et al.* PANTHER: a library of protein families and subfamilies indexed by function. *Genome Res* **13**, 2129-2141 (2003).

6. Bindea, G.*, et al.* ClueGO: a Cytoscape plug-in to decipher functionally grouped gene ontology and pathway annotation networks. *Bioinformatics* **25**, 1091-1093 (2009).

7. Kramer, A., Green, J., Pollard, J., Jr. & Tugendreich, S. Causal analysis approaches in Ingenuity Pathway Analysis. *Bioinformatics* **30**, 523-530 (2014).

8. Xie, C.*, et al.* KOBAS 2.0: a web server for annotation and identification of enriched pathways and diseases. *Nucleic Acids Res* **39**, W316-322 (2011).

9. Chen, J., Bardes, E.E., Aronow, B.J. & Jegga, A.G. ToppGene Suite for gene list enrichment analysis and candidate gene prioritization. *Nucleic Acids Res* **37**, W305-W311 (2009).

10. Merico, D., Isserlin, R., Stueker, O., Emili, A. & Bader, G.D. Enrichment map: a network-based method for gene-set enrichment visualization and interpretation. *PLoS One* **5**, e13984 (2010).

11. Zhou, Y.*, et al.* Metascape provides a biologist-oriented resource for the analysis of systems-level datasets. *Nat Commun* **10**, 1523 (2019).

12. Zhang, B., Kirov, S. & Snoddy, J. WebGestalt: an integrated system for exploring gene sets in various biological contexts. *Nucleic Acids Res* **33**, W741-748 (2005).

13. Ernst, J. & Bar-Joseph, Z. STEM: a tool for the analysis of short time series gene expression data. *BMC Bioinformatics* **7**, 191 (2006).

14. Reimand, J.*, et al.* g:Profiler-a web server for functional interpretation of gene lists (2016 update). *Nucleic Acids Res* **44**, W83-W89 (2016).

15. Luo, W., Friedman, M.S., Shedden, K., Hankenson, K.D. & Woolf, P.J. GAGE: generally applicable gene set enrichment for pathway analysis. *BMC Bioinformatics* **10**, 161 (2009).

16. Kim, S.Y. & Volsky, D.J. PAGE: parametric analysis of gene set enrichment. *BMC Bioinformatics* **6**, 144 (2005).

17. Carmona-Saez, P., Chagoyen, M., Tirado, F., Carazo, J.M. & Pascual-Montano, A. GENECODIS: a web-based tool for finding significant concurrent annotations in gene lists. *Genome Biol* **8**, R3 (2007).

18. Backes, C.*, et al.* GeneTrail--advanced gene set enrichment analysis. *Nucleic Acids Res* **35**, W186-192 (2007).

19. Medina, I.*, et al.* Babelomics: an integrative platform for the analysis of transcriptomics, proteomics and genomic data with advanced functional profiling. *Nucleic Acids Res* **38**, W210-213 (2010).

20. Usadel, B.*, et al.* PageMan: an interactive ontology tool to generate, display, and annotate overview graphs for profiling experiments. *BMC Bioinformatics* **7**, 535 (2006).

21. Lee, H.K., Braynen, W., Keshav, K. & Pavlidis, P. ErmineJ: tool for functional analysis of gene expression data sets. *BMC Bioinformatics* **6**, 269 (2005).

22. Glaab, E., Baudot, A., Krasnogor, N., Schneider, R. & Valencia, A. EnrichNet: network-based gene set enrichment analysis. *Bioinformatics* **28**, i451-i457 (2012).

23. Yu, G., Wang, L.G., Yan, G.R. & He, Q.Y. DOSE: an R/Bioconductor package for disease ontology semantic and enrichment analysis. *Bioinformatics* **31**, 608-609 (2015).

24. Newman, J.C. & Weiner, A.M. L2L: a simple tool for discovering the hidden significance in microarray expression data. *Genome Biol* **6**, R81 (2005).

25. Hur, J., Schuyler, A.D., States, D.J. & Feldman, E.L. SciMiner: web-based literature mining tool for target identification and functional enrichment analysis. *Bioinformatics* **25**, 838-840 (2009).

26. Frijters, R.*, et al.* CoPub: a literature-based keyword enrichment tool for microarray data analysis. *Nucleic Acids Res* **36**, W406-410 (2008).

27. Prifti, E., Zucker, J.D., Clement, K. & Henegar, C. FunNet: an integrative tool for exploring transcriptional interactions. *Bioinformatics* **24**, 2636-2638 (2008).

28. Chung, H.J., Kim, M., Park, C.H., Kim, J. & Kim, J.H. ArrayXPath: mapping and visualizing microarray gene-expression data with integrated biological pathway resources using Scalable Vector Graphics. *Nucleic Acids Res* **32**, W460-464 (2004).

29. Antonov, A.V., Schmidt, T., Wang, Y. & Mewes, H.W. ProfCom: a web tool for profiling the complex functionality of gene groups identified from high-throughput data. *Nucleic Acids Res* **36**, W347-351 (2008).

30. Freudenberg, J.M., Joshi, V.K., Hu, Z. & Medvedovic, M. CLEAN: CLustering Enrichment ANalysis. *BMC Bioinformatics* **10**, 234 (2009).

31. D'Andrea, D., Grassi, L., Mazzapioda, M. & Tramontano, A. FIDEA: a server for the functional interpretation of differential expression analysis. *Nucleic Acids Res* **41**, W84-88 (2013).

32. Tan, C.M., Chen, E.Y., Dannenfelser, R., Clark, N.R. & Ma'ayan, A. Network2Canvas: network visualization on a canvas with enrichment analysis. *Bioinformatics* **29**, 1872-1878 (2013).

33. Deng, Y., Gao, L., Wang, B. & Guo, X. HPOSim: an R package for phenotypic similarity measure and enrichment analysis based on the human phenotype ontology. *PLoS One* **10**, e0115692 (2015).

34. Clark, N.R.*, et al.* Principal Angle Enrichment Analysis (PAEA): Dimensionally Reduced Multivariate Gene Set Enrichment Analysis Tool. *Proceedings (IEEE Int Conf Bioinformatics Biomed)* **2015**, 256-262 (2015).

35. Federico, A. & Monti, S. hypeR: an R package for geneset enrichment workflows. *Bioinformatics* **36**, 1307-1308 (2020).

36. Farahmand, S., O'Connor, C., Macoska, J.A. & Zarringhalam, K. Causal Inference Engine: a platform for directional gene set enrichment analysis and inference of active transcriptional regulators. *Nucleic Acids Res* **47**, 11563-11573 (2019).

37. Feng, G., Du, P., Kibbe, W.A. & Lin, S. GeneAnswers, integrated interpretation of genes. *Bioinformatics* **25**, i63-68 (2010).

38. Maere, S., Heymans, K. & Kuiper, M. BiNGO: a Cytoscape plugin to assess overrepresentation of gene ontology categories in biological networks. *Bioinformatics* **21**, 3448-3449 (2005).

39. Ye, J.*, et al.* WEGO: a web tool for plotting GO annotations. *Nucleic Acids Res* **34**, W293-297 (2006).

40. Tian, T.*, et al.* agriGO v2.0: a GO analysis toolkit for the agricultural community, 2017 update. *Nucleic Acids Res* **45**, W122-W129 (2017).

41. Falcon, S. & Gentleman, R. Using GOstats to test gene lists for GO term association. *Bioinformatics* **23**, 257-258 (2007).

42. Boyle, E.I.*, et al.* GO::TermFinder--open source software for accessing Gene Ontology information and finding significantly enriched Gene Ontology terms associated with a list of genes. *Bioinformatics* **20**, 3710-3715 (2004).

43. Zeeberg, B.R.*, et al.* GoMiner: a resource for biological interpretation of genomic and proteomic data. *Genome Biol* **4**, R28 (2003).

44. Beissbarth, T. & Speed, T.P. GOstat: find statistically overrepresented Gene Ontologies within a group of genes. *Bioinformatics* **20**, 1464-1465 (2004).

45. Alexa, A. & Rahnenfuhrer, J. topGO: enrichment analysis for gene ontology. *R package version* **2**, 2010 (2010).

46. Zheng, Q. & Wang, X.J. GOEAST: a web-based software toolkit for Gene Ontology enrichment analysis. *Nucleic Acids Res* **36**, W358-363 (2008).

47. Berriz, G.F., King, O.D., Bryant, B., Sander, C. & Roth, F.P. Characterizing gene sets with FuncAssociate. *Bioinformatics* **19**, 2502-2504 (2003).

48. Khatri, P., Draghici, S., Ostermeier, G.C. & Krawetz, S.A. Profiling gene expression using onto-express. *Genomics* **79**, 266-270 (2002).

49. Bluthgen, N.*, et al.* Biological profiling of gene groups utilizing Gene Ontology. *Genome Inform* **16**, 106-115 (2005).

50. Frohlich, H., Speer, N., Poustka, A. & Beissbarth, T. GOSim--an R-package for computation of information theoretic GO similarities between terms and gene products. *BMC Bioinformatics* **8**, 166 (2007).

51. Zhou, X. & Su, Z. EasyGO: Gene Ontology-based annotation and functional enrichment analysis tool for agronomical species. *BMC Genomics* **8**, 246 (2007).

52. Zhong, S.*, et al.* GoSurfer: a graphical interactive tool for comparative analysis of large gene sets in Gene Ontology space. *Appl Bioinformatics* **3**, 261-264 (2004).

53. Beisvag, V.*, et al.* GeneTools--application for functional annotation and statistical hypothesis testing. *BMC Bioinformatics* **7**, 470 (2006).

54. Ge, S.X., Jung, D. & Yao, R. ShinyGO: a graphical gene-set enrichment tool for animals and plants. *Bioinformatics* **36**, 2628-2629 (2020).

55. Sealfon, R.S., Hibbs, M.A., Huttenhower, C., Myers, C.L. & Troyanskaya, O.G. GOLEM: an interactive graph-based gene-ontology navigation and analysis tool. *BMC Bioinformatics* **7**, 443 (2006).

56. Sun, H., Fang, H., Chen, T., Perkins, R. & Tong, W. GOFFA: gene ontology for functional analysis--a FDA gene ontology tool for analysis of genomic and proteomic data. *BMC Bioinformatics* **7 Suppl 2**, S23 (2006).

57. Vencio, R.Z., Koide, T., Gomes, S.L. & Pereira, C.A. BayGO: Bayesian analysis of ontology term enrichment in microarray data. *BMC Bioinformatics* **7**, 86 (2006).

58. Nam, D.*, et al.* ADGO: analysis of differentially expressed gene sets using composite GO annotation. *Bioinformatics* **22**, 2249-2253 (2006).

59. Scheer, M.*, et al.* JProGO: a novel tool for the functional interpretation of prokaryotic microarray data using Gene Ontology information. *Nucleic Acids Res* **34**, W510-515 (2006).

60. Lee, J.S., Katari, G. & Sachidanandam, R. GObar: a gene ontology based analysis and visualization tool for gene sets. *BMC Bioinformatics* **6**, 189 (2005).

61. Wrobel, G., Chalmel, F. & Primig, M. goCluster integrates statistical analysis and functional interpretation of microarray expression data. *Bioinformatics* **21**, 3575-3577 (2005).

62. Henegar, C.*, et al.* Clustering biological annotations and gene expression data to identify putatively co-regulated biological processes. *J Bioinform Comput Biol* **4**, 833-852 (2006).

63. Weng, M.P. & Liao, B.Y. MamPhEA: a web tool for mammalian phenotype enrichment analysis. *Bioinformatics* **26**, 2212-2213 (2010).

64. Vencio, R.Z. & Shmulevich, I. ProbCD: enrichment analysis accounting for categorization uncertainty. *BMC Bioinformatics* **8**, 383 (2007).

65. Kim, J., Yoon, S. & Nam, D. netGO: R-Shiny package for network-integrated pathway enrichment analysis. *Bioinformatics* **36**, 3283-3285 (2020).

66. Conesa, A.*, et al.* Blast2GO: a universal tool for annotation, visualization and analysis in functional genomics research. *Bioinformatics* **21**, 3674-3676 (2005).

67. Komurov, K., Dursun, S., Erdin, S. & Ram, P.T. NetWalker: a contextual network analysis tool for functional genomics. *BMC Genomics* **13**, 282 (2012).

68. Sales, G., Calura, E., Martini, P. & Romualdi, C. Graphite Web: Web tool for gene set analysis exploiting pathway topology. *Nucleic Acids Res* **41**, W89-97 (2013).

69. Hale, M.L., Thapa, I. & Ghersi, D. FunSet: an open-source software and web server for performing and displaying Gene Ontology enrichment analysis. *BMC Bioinformatics* **20**, 359 (2019).

70. Szklarczyk, D.*, et al.* The STRING database in 2017: quality-controlled protein–protein association networks, made broadly accessible. *Nucleic acids research*, gkw937 (2016).

71. Keshava Prasad, T.*, et al.* Human protein reference database—2009 update. *Nucleic acids research* **37**, D767-D772 (2009).

72. Orchard, S.*, et al.* The MIntAct project—IntAct as a common curation platform for 11 molecular interaction databases. *Nucleic acids research* **42**, D358-D363 (2014).

73. Alfarano, C.*, et al.* The biomolecular interaction network database and related tools 2005 update. *Nucleic acids research* **33**, D418-D424 (2005).

74. Xenarios, I.*, et al.* DIP: the database of interacting proteins. *Nucleic acids research* **28**, 289-291 (2000).

75. Oughtred, R.*, et al.* The BioGRID interaction database: 2019 update. *Nucleic acids research* **47**, D529-D541 (2019).

76. Chatr-Aryamontri, A.*, et al.* MINT: the Molecular INTeraction database. *Nucleic acids research* **35**, D572-D574 (2007).

77. Breuer, K.*, et al.* InnateDB: systems biology of innate immunity and beyond—recent updates and continuing curation. *Nucleic acids research* **41**, D1228-D1233 (2013).

78. Luck, K.*, et al.* A reference map of the human binary protein interactome. *Nature* **580**, 402-408 (2020).

79. Orchard, S.*, et al.* Protein interaction data curation: the International Molecular Exchange (IMEx) consortium. *Nature methods* **9**, 345-350 (2012).

80. Li, T.*, et al.* A scored human protein–protein interaction network to catalyze genomic interpretation. *Nature methods* **14**, 61-64 (2017).

81. Pratt, D.*, et al.* NDEx, the network data exchange. *Cell systems* **1**, 302-305 (2015).

82. Kotlyar, M., Pastrello, C., Sheahan, N. & Jurisica, I. Integrated interactions database: tissue-specific view of the human and model organism interactomes. *Nucleic acids research* **44**, D536-D541 (2016).

83. Licata, L.*, et al.* SIGNOR 2.0, the SIGnaling network open resource 2.0: 2019 update. *Nucleic acids research* **48**, D504-D510 (2020).

84. Clerc, O.*, et al.* MatrixDB: integration of new data with a focus on glycosaminoglycan interactions. *Nucleic acids research* **47**, D376-D381 (2019).

85. Niu, Y., Otasek, D. & Jurisica, I. Evaluation of linguistic features useful in extraction of interactions from PubMed; application to annotating known, high-throughput and predicted interactions in I2D. *Bioinformatics* **26**, 111-119 (2010).

86. Elkon, R.*, et al.* SPIKE–a database, visualization and analysis tool of cellular signaling pathways. *BMC bioinformatics* **9**, 1-15 (2008).

87. Fornes, O.*, et al.* JASPAR 2020: update of the open-access database of transcription factor binding profiles. *Nucleic acids research* **48**, D87-D92 (2020).

88. Lachmann, A.*, et al.* ChEA: transcription factor regulation inferred from integrating genome-wide ChIP-X experiments. *Bioinformatics* **26**, 2438-2444 (2010).

89. Hu, H.*, et al.* AnimalTFDB 3.0: a comprehensive resource for annotation and prediction of animal transcription factors. *Nucleic acids research* **47**, D33-D38 (2019).

90. Jiang, C., Xuan, Z., Zhao, F. & Zhang, M.Q. TRED: a transcriptional regulatory element database, new entries and other development. *Nucleic acids research* **35**, D137-D140 (2007).

91. Liu, Z.-P., Wu, C., Miao, H. & Wu, H. RegNetwork: an integrated database of transcriptional and post-transcriptional regulatory networks in human and mouse. *Database* **2015**(2015).

92. Lescot, M.*, et al.* PlantCARE, a database of plant cis-acting regulatory elements and a portal to tools for in silico analysis of promoter sequences. *Nucleic Acids Res* **30**, 325-327 (2002).

93. Lewis, B.P., Burge, C.B. & Bartel, D.P. Conserved seed pairing, often flanked by adenosines, indicates that thousands of human genes are microRNA targets. *cell* **120**, 15-20 (2005).

94. Kozomara, A., Birgaoanu, M. & Griffiths-Jones, S. miRBase: from microRNA sequences to function. *Nucleic acids research* **47**, D155-D162 (2019).

95. Chen, Y. & Wang, X. miRDB: an online database for prediction of functional microRNA targets. *Nucleic acids research* **48**, D127-D131 (2020).

96. Xiao, F.*, et al.* miRecords: an integrated resource for microRNA–target interactions. *Nucleic acids research* **37**, D105-D110 (2009).

97. Krüger, J. & Rehmsmeier, M. RNAhybrid: microRNA target prediction easy, fast and flexible. *Nucleic acids research* **34**, W451-W454 (2006).

98. Huang, H.-Y.*, et al.* miRTarBase 2020: updates to the experimentally validated microRNA–target interaction database. *Nucleic acids research* **48**, D148-D154 (2020).

99. Jiang, Q.*, et al.* miR2Disease: a manually curated database for microRNA deregulation in human disease. *Nucleic acids research* **37**, D98-D104 (2009).

100. Karagkouni, D.*, et al.* DIANA-TarBase v8: a decade-long collection of experimentally supported miRNA–gene interactions. *Nucleic acids research* **46**, D239-D245 (2018).

101. Tokar, T.*, et al.* mirDIP 4.1—integrative database of human microRNA target predictions. *Nucleic acids research* **46**, D360-D370 (2018).

102. Bandyopadhyay, S. & Mitra, R. TargetMiner: microRNA target prediction with systematic identification of tissue-specific negative examples. *Bioinformatics* **25**, 2625-2631 (2009).

103. Moore, J.E.*, et al.* Expanded encyclopaedias of DNA elements in the human and mouse genomes. *Nature* **583**, 699-710 (2020).

104. Li, J.-H., Liu, S., Zhou, H., Qu, L.-H. & Yang, J.-H. starBase v2. 0: decoding miRNA-ceRNA, miRNA-ncRNA and protein–RNA interaction networks from large-scale CLIP-Seq data. *Nucleic acids research* **42**, D92-D97 (2014).

105. Jeggari, A., Marks, D.S. & Larsson, E. miRcode: a map of putative microRNA target sites in the long non-coding transcriptome. *Bioinformatics* **28**, 2062-2063 (2012).

106. Paraskevopoulou, M.D.*, et al.* DIANA-LncBase v2: indexing microRNA targets on non-coding transcripts. *Nucleic acids research* **44**, D231-D238 (2016).

107. Türei, D., Korcsmáros, T. & Saez-Rodriguez, J. OmniPath: guidelines and gateway for literature-curated signaling pathway resources. *Nature methods* **13**, 966-967 (2016).

108. Wang, P.*, et al.* miRSponge: a manually curated database for experimentally supported miRNA sponges and ceRNAs. *Database* **2015**(2015).

109. Sarver, A.L. & Subramanian, S. Competing endogenous RNA database. *Bioinformation* **8**, 731 (2012).

110. Wang, P.*, et al.* LncACTdb 2.0: an updated database of experimentally supported ceRNA interactions curated from low-and high-throughput experiments. *Nucleic acids research* **47**, D121-D127 (2019).

111. Yuan, C.*, et al.* PceRBase: a database of plant competing endogenous RNA. *Nucleic acids research* **45**, D1009-D1014 (2017).

112. Franz, M.*, et al.* GeneMANIA update 2018. *Nucleic Acids Res* **46**, W60-W64 (2018).

113. Zhou, G.*, et al.* NetworkAnalyst 3.0: a visual analytics platform for comprehensive gene expression profiling and meta-analysis. *Nucleic Acids Res* **47**, W234-W241 (2019).

114. Herwig, R., Hardt, C., Lienhard, M. & Kamburov, A. Analyzing and interpreting genome data at the network level with ConsensusPathDB. *Nat Protoc* **11**, 1889-1907 (2016).

115. Janky, R.*, et al.* iRegulon: from a gene list to a gene regulatory network using large motif and track collections. *PLoS Comput Biol* **10**, e1003731 (2014).

116. Brown, K.R.*, et al.* NAViGaTOR: Network Analysis, Visualization and Graphing Toronto. *Bioinformatics* **25**, 3327-3329 (2009).

117. Antonov, A.V. BioProfiling.de: analytical web portal for high-throughput cell biology. *Nucleic Acids Res* **39**, W323-327 (2011).

118. Schmitt, T., Ogris, C. & Sonnhammer, E.L. FunCoup 3.0: database of genome-wide functional coupling networks. *Nucleic Acids Res* **42**, D380-388 (2014).

119. Kern, F.*, et al.* miRTargetLink 2.0—interactive miRNA target gene and target pathway networks. *Nucleic Acids Research* **49**, W409-W416 (2021).

120. Minguez, P., Gotz, S., Montaner, D., Al-Shahrour, F. & Dopazo, J. SNOW, a web-based tool for the statistical analysis of protein-protein interaction networks. *Nucleic Acids Res* **37**, W109-114 (2009).

121. Xie, G.Y.*, et al.* FFLtool: a web server for transcription factor and miRNA feed forward loop analysis in human. *Bioinformatics* **36**, 2605-2607 (2020).

122. Wei, C.-H., Allot, A., Leaman, R. & Lu, Z. PubTator central: automated concept annotation for biomedical full text articles. *Nucleic acids research* **47**, W587-W593 (2019).

123. Yang, H., Robinson, P.N. & Wang, K. Phenolyzer: phenotype-based prioritization of candidate genes for human diseases. *Nature methods* **12**, 841-843 (2015).

124. Liekens, A.M.*, et al.* BioGraph: unsupervised biomedical knowledge discovery via automated hypothesis generation. *Genome biology* **12**, 1-12 (2011).

125. Oesper, L., Merico, D., Isserlin, R. & Bader, G.D. WordCloud: a Cytoscape plugin to create a visual semantic summary of networks. *Source code for biology and medicine* **6**, 1-4 (2011).

126. Lee, S.*, et al.* BEST: next-generation biomedical entity search tool for knowledge discovery from biomedical literature. *PloS one* **11**, e0164680 (2016).

127. Huang, H.*, et al.* iPTMnet: an integrated resource for protein post-translational modification network discovery. *Nucleic acids research* **46**, D542-D550 (2018).

128. Baroukh, C., Jenkins, S.L., Dannenfelser, R. & Ma'Ayan, A. Genes2WordCloud: a quick way to identify biological themes from gene lists and free text. *Source code for biology and medicine* **6**, 1-5 (2011).

129. Islamaj, R., Kwon, D., Kim, S. & Lu, Z. TeamTat: a collaborative text annotation tool. *Nucleic acids research* **48**, W5-W11 (2020).

130. Peng, C.*, et al.* CADA: phenotype-driven gene prioritization based on a case-enriched knowledge graph. *NAR genomics and bioinformatics* **3**, lqab078 (2021).

131. Dogan, T.*, et al.* CROssBAR: comprehensive resource of biomedical relations with knowledge graph representations. *Nucleic Acids Res* **49**, e96 (2021).

132. Subramanian, A.*, et al.* A Next Generation Connectivity Map: L1000 Platform and the First 1,000,000 Profiles. *Cell* **171**, 1437-1452 e1417 (2017).

133. Emig, D.*, et al.* Drug target prediction and repositioning using an integrated network-based approach. *PLoS One* **8**, e60618 (2013).

134. Wang, Z.*, et al.* Extraction and analysis of signatures from the Gene Expression Omnibus by the crowd. *Nat Commun* **7**, 12846 (2016).

135. Duan, Q.*, et al.* L1000CDS(2): LINCS L1000 characteristic direction signatures search engine. *NPJ Syst Biol Appl* **2**(2016).

136. Smirnov, P.*, et al.* PharmacoGx: an R package for analysis of large pharmacogenomic datasets. *Bioinformatics* **32**, 1244-1246 (2016).

137. Huang, L.*, et al.* DrugComboRanker: drug combination discovery based on target network analysis. *Bioinformatics* **30**, i228-i236 (2014).

138. Wang, Z., Clark, N.R. & Ma’ayan, A. Drug-induced adverse events prediction with the LINCS L1000 data. *Bioinformatics* **32**, 2338-2345 (2016).

139. Wang, Z., Lachmann, A., Keenan, A.B. & Ma’ayan, A. L1000FWD: fireworks visualization of drug-induced transcriptomic signatures. *Bioinformatics* **34**, 2150-2152 (2018).

140. Carrella, D.*, et al.* Mantra 2.0: an online collaborative resource for drug mode of action and repurposing by network analysis. *Bioinformatics* **30**, 1787-1788 (2014).

141. Napolitano, F., Sirci, F., Carrella, D. & di Bernardo, D. Drug-set enrichment analysis: a novel tool to investigate drug mode of action. *Bioinformatics* **32**, 235-241 (2016).

142. Pacini, C.*, et al.* DvD: An R/Cytoscape pipeline for drug repurposing using public repositories of gene expression data. *Bioinformatics* **29**, 132-134 (2013).

143. Jia, Z.*, et al.* Cogena, a novel tool for co-expressed gene-set enrichment analysis, applied to drug repositioning and drug mode of action discovery. *BMC Genomics* **17**, 414 (2016).

144. Napolitano, F.*, et al.* gene2drug: a computational tool for pathway-based rational drug repositioning. *Bioinformatics* **34**, 1498-1505 (2018).

145. Lee, B.K.*, et al.* DeSigN: connecting gene expression with therapeutics for drug repurposing and development. *BMC Genomics* **18**, 934 (2017).

146. Brown, A.S., Kong, S.W., Kohane, I.S. & Patel, C.J. ksRepo: a generalized platform for computational drug repositioning. *BMC Bioinformatics* **17**, 78 (2016).

147. Pilarczyk, M.*, et al.* Connecting omics signatures of diseases, drugs, and mechanisms of actions with iLINCS. *BioRxiv*, 826271 (2020).

148. Li, F.*, et al.* GeneExpressionSignature: an R package for discovering functional connections using gene expression signatures. *OMICS* **17**, 116-118 (2013).

149. Yu, H.*, et al.* Prediction of drugs having opposite effects on disease genes in a directed network. *BMC Syst Biol* **10 Suppl 1**, 2 (2016).

150. Evangelista, J.E.*, et al.* SigCom LINCS: data and metadata search engine for a million gene expression signatures. *Nucleic Acids Research* (2022).

151. Newman, A.M.*, et al.* Robust enumeration of cell subsets from tissue expression profiles. *Nat Methods* **12**, 453-457 (2015).

152. Yoshihara, K.*, et al.* Inferring tumour purity and stromal and immune cell admixture from expression data. *Nat Commun* **4**, 2612 (2013).

153. Aran, D., Hu, Z. & Butte, A.J. xCell: digitally portraying the tissue cellular heterogeneity landscape. *Genome Biol* **18**, 220 (2017).

154. Li, T.*, et al.* TIMER2.0 for analysis of tumor-infiltrating immune cells. *Nucleic Acids Res* **48**, W509-W514 (2020).

155. Becht, E.*, et al.* Estimating the population abundance of tissue-infiltrating immune and stromal cell populations using gene expression. *Genome Biol* **17**, 218 (2016).

156. Newman, A.M.*, et al.* Determining cell type abundance and expression from bulk tissues with digital cytometry. *Nat Biotechnol* **37**, 773-782 (2019).

157. Baron, M.*, et al.* A Single-Cell Transcriptomic Map of the Human and Mouse Pancreas Reveals Inter- and Intra-cell Population Structure. *Cell Syst* **3**, 346-360 e344 (2016).

158. Racle, J., de Jonge, K., Baumgaertner, P., Speiser, D.E. & Gfeller, D. Simultaneous enumeration of cancer and immune cell types from bulk tumor gene expression data. *Elife* **6**(2017).

159. Finotello, F.*, et al.* Molecular and pharmacological modulators of the tumor immune contexture revealed by deconvolution of RNA-seq data. *Genome Med* **11**, 34 (2019).

160. Abbas, A.R., Wolslegel, K., Seshasayee, D., Modrusan, Z. & Clark, H.F. Deconvolution of blood microarray data identifies cellular activation patterns in systemic lupus erythematosus. *PLoS One* **4**, e6098 (2009).

161. Monaco, G.*, et al.* RNA-Seq Signatures Normalized by mRNA Abundance Allow Absolute Deconvolution of Human Immune Cell Types. *Cell Rep* **26**, 1627-1640 e1627 (2019).

162. Miao, Y.R.*, et al.* ImmuCellAI: A Unique Method for Comprehensive T-Cell Subsets Abundance Prediction and its Application in Cancer Immunotherapy. *Adv Sci (Weinh)* **7**, 1902880 (2020).

163. Wang, X., Park, J., Susztak, K., Zhang, N.R. & Li, M. Bulk tissue cell type deconvolution with multi-subject single-cell expression reference. *Nat Commun* **10**, 380 (2019).

164. Gong, T. & Szustakowski, J.D. DeconRNASeq: a statistical framework for deconvolution of heterogeneous tissue samples based on mRNA-Seq data. *Bioinformatics* **29**, 1083-1085 (2013).

165. Schelker, M.*, et al.* Estimation of immune cell content in tumour tissue using single-cell RNA-seq data. *Nat Commun* **8**, 2032 (2017).

166. Zhong, Y., Wan, Y.W., Pang, K., Chow, L.M. & Liu, Z. Digital sorting of complex tissues for cell type-specific gene expression profiles. *BMC Bioinformatics* **14**, 89 (2013).

167. Foroutan, M.*, et al.* Single sample scoring of molecular phenotypes. *BMC Bioinformatics* **19**, 404 (2018).

168. Gong, T.*, et al.* Optimal deconvolution of transcriptional profiling data using quadratic programming with application to complex clinical blood samples. *PLoS One* **6**, e27156 (2011).

169. Qiao, W.*, et al.* PERT: a method for expression deconvolution of human blood samples from varied microenvironmental and developmental conditions. *PLoS Comput Biol* **8**, e1002838 (2012).

170. Chen, Z.*, et al.* Inference of immune cell composition on the expression profiles of mouse tissue. *Sci Rep* **7**, 40508 (2017).

171. Vallania, F.*, et al.* Leveraging heterogeneity across multiple datasets increases cell-mixture deconvolution accuracy and reduces biological and technical biases. *Nat Commun* **9**, 4735 (2018).

172. Altboum, Z.*, et al.* Digital cell quantification identifies global immune cell dynamics during influenza infection. *Mol Syst Biol* **10**, 720 (2014).

173. Dong, M.*, et al.* SCDC: bulk gene expression deconvolution by multiple single-cell RNA sequencing references. *Brief Bioinform* **22**, 416-427 (2021).

174. Onuchic, V.*, et al.* Epigenomic Deconvolution of Breast Tumors Reveals Metabolic Coupling between Constituent Cell Types. *Cell Rep* **17**, 2075-2086 (2016).

175. Liebner, D.A., Huang, K. & Parvin, J.D. MMAD: microarray microdissection with analysis of differences is a computational tool for deconvoluting cell type-specific contributions from tissue samples. *Bioinformatics* **30**, 682-689 (2014).

176. Frishberg, A.*, et al.* Cell composition analysis of bulk genomics using single-cell data. *Nat Methods* **16**, 327-332 (2019).

177. Wang, N.*, et al.* Mathematical modelling of transcriptional heterogeneity identifies novel markers and subpopulations in complex tissues. *Sci Rep* **6**, 18909 (2016).

178. Li, Y. & Xie, X. A mixture model for expression deconvolution from RNA-seq in heterogeneous tissues. *BMC Bioinformatics* **14 Suppl 5**, S11 (2013).

179. Wang, Z.*, et al.* Transcriptome Deconvolution of Heterogeneous Tumor Samples with Immune Infiltration. *iScience* **9**, 451-460 (2018).

180. Hunt, G.J., Freytag, S., Bahlo, M. & Gagnon-Bartsch, J.A. dtangle: accurate and robust cell type deconvolution. *Bioinformatics* **35**, 2093-2099 (2019).

181. Hao, Y., Yan, M., Heath, B.R., Lei, Y.L. & Xie, Y. Fast and robust deconvolution of tumor infiltrating lymphocyte from expression profiles using least trimmed squares. *PLoS Comput Biol* **15**, e1006976 (2019).

182. Li, Z. & Wu, H. TOAST: improving reference-free cell composition estimation by cross-cell type differential analysis. *Genome Biol* **20**, 190 (2019).

183. Kang, K.*, et al.* CDSeq: A novel complete deconvolution method for dissecting heterogeneous samples using gene expression data. *PLoS Comput Biol* **15**, e1007510 (2019).

184. Shannon, C.P.*, et al.* Two-stage, in silico deconvolution of the lymphocyte compartment of the peripheral whole blood transcriptome in the context of acute kidney allograft rejection. *PLoS One* **9**, e95224 (2014).

185. Du, R., Carey, V. & Weiss, S.T. deconvSeq: deconvolution of cell mixture distribution in sequencing data. *Bioinformatics* **35**, 5095-5102 (2019).

186. Yu, X., Chen, Y.A., Conejo-Garcia, J.R., Chung, C.H. & Wang, X. Estimation of immune cell content in tumor using single-cell RNA-seq reference data. *BMC Cancer* **19**, 715 (2019).

187. Torroja, C. & Sanchez-Cabo, F. Digitaldlsorter: Deep-Learning on scRNA-Seq to Deconvolute Gene Expression Data. *Front Genet* **10**, 978 (2019).

188. Wang, L.*, et al.* A reference profile-free deconvolution method to infer cancer cell-intrinsic subtypes and tumor-type-specific stromal profiles. *Genome Med* **12**, 24 (2020).

189. Chen, S.H.*, et al.* A gene profiling deconvolution approach to estimating immune cell composition from complex tissues. *BMC Bioinformatics* **19**, 154 (2018).

190. Ogundijo, O.E. & Wang, X. A sequential Monte Carlo approach to gene expression deconvolution. *PLoS One* **12**, e0186167 (2017).

191. Chiu, Y.J., Hsieh, Y.H. & Huang, Y.H. Improved cell composition deconvolution method of bulk gene expression profiles to quantify subsets of immune cells. *BMC Med Genomics* **12**, 169 (2019).

192. Tang, D., Park, S. & Zhao, H. NITUMID: Nonnegative matrix factorization-based Immune-TUmor MIcroenvironment Deconvolution. *Bioinformatics* **36**, 1344-1350 (2020).

193. Danziger, S.A.*, et al.* ADAPTS: Automated deconvolution augmentation of profiles for tissue specific cells. *PLoS One* **14**, e0224693 (2019).

194. Sun, X., Sun, S. & Yang, S. An Efficient and Flexible Method for Deconvoluting Bulk RNA-Seq Data with Single-Cell RNA-Seq Data. *Cells* **8**(2019).

195. Dimitrakopoulou, K., Wik, E., Akslen, L.A. & Jonassen, I. Deblender: a semi-/unsupervised multi-operational computational method for complete deconvolution of expression data from heterogeneous samples. *BMC Bioinformatics* **19**, 408 (2018).

196. Qi, L.*, et al.* Deconvolution of the gene expression profiles of valuable banked blood specimens for studying the prognostic values of altered peripheral immune cell proportions in cancer patients. *PLoS One* **9**, e100934 (2014).

197. Fernández, E.A.*, et al.* MIXTURE: an improved algorithm for immune tumor microenvironment estimation based on gene expression data. *BioRxiv*, 726562 (2019).

198. Dong, L., Kollipara, A., Darville, T., Zou, F. & Zheng, X. Semi-CAM: A semi-supervised deconvolution method for bulk transcriptomic data with partial marker gene information. *Sci Rep* **10**, 5434 (2020).

199. Schon, M.*, et al.* DTD: An R Package for Digital Tissue Deconvolution. *J Comput Biol* **27**, 386-389 (2020).

200. Zaslavsky, M., Novik, J.B., Chang, E. & Hammerbacher, J. Infino: a Bayesian hierarchical model improves estimates of immune infiltration into tumor microenvironment. *bioRxiv*, 221671 (2017).

201. Bergen, V., Lange, M., Peidli, S., Wolf, F.A. & Theis, F.J. Generalizing RNA velocity to transient cell states through dynamical modeling. *Nat Biotechnol* **38**, 1408-1414 (2020).

202. Street, K.*, et al.* Slingshot: cell lineage and pseudotime inference for single-cell transcriptomics. *BMC Genomics* **19**, 477 (2018).

203. Lange, M.*, et al.* CellRank for directed single-cell fate mapping. *Nat Methods* **19**, 159-170 (2022).

204. Atta, L., Sahoo, A. & Fan, J. VeloViz: RNA velocity informed embeddings for visualizing cellular trajectories. *Bioinformatics* **38**, 391-396 (2021).

205. Weng, G., Kim, J. & Won, K.J. VeTra: a tool for trajectory inference based on RNA velocity. *Bioinformatics* **37**, 3509-3513 (2021).

206. Conard, A.M.*, et al.* TIMEOR: a web-based tool to uncover temporal regulatory mechanisms from multi-omics data. *Nucleic Acids Res* **49**, W641-W653 (2021).

207. Gao, M., Qiao, C. & Huang, Y. UniTVelo: temporally unified RNA velocity reinforces single-cell trajectory inference. *Nat Commun* **13**, 6586 (2022).

208. Zheng, Z.*, et al.* TIPS: trajectory inference of pathway significance through pseudotime comparison for functional assessment of single-cell RNAseq data. *Brief Bioinform* **22**(2021).

209. Wang, X. & Zheng, J. Velo-Predictor: an ensemble learning pipeline for RNA velocity prediction. *BMC Bioinformatics* **22**, 419 (2021).

210. Senra, D., Guisoni, N. & Diambra, L. ORIGINS: A protein network-based approach to quantify cell pluripotency from scRNA-seq data. *MethodsX* **9**, 101778 (2022).

211. Harding, S.D.*, et al.* The IUPHAR/BPS Guide to PHARMACOLOGY in 2018: updates and expansion to encompass the new guide to IMMUNOPHARMACOLOGY. *Nucleic acids research* **46**, D1091-D1106 (2018).

212. Efremova, M., Vento-Tormo, M., Teichmann, S.A. & Vento-Tormo, R. CellPhoneDB: inferring cell–cell communication from combined expression of multi-subunit ligand–receptor complexes. *Nature protocols* **15**, 1484-1506 (2020).

213. Lizio, M.*, et al.* Gateways to the FANTOM5 promoter level mammalian expression atlas. *Genome Biol* **16**, 22 (2015).

214. Ramilowski, J.A.*, et al.* A draft network of ligand–receptor-mediated multicellular signalling in human. *Nature communications* **6**, 1-12 (2015).

215. Armingol, E., Officer, A., Harismendy, O. & Lewis, N.E. Deciphering cell-cell interactions and communication from gene expression. *Nat Rev Genet* **22**, 71-88 (2021).

216. Graeber, T.G. & Eisenberg, D. Bioinformatic identification of potential autocrine signaling loops in cancers from gene expression profiles. *Nature genetics* **29**, 295-300 (2001).

217. Hodge, M.R.*, et al.* ConnectomeDB—sharing human brain connectivity data. *Neuroimage* **124**, 1102-1107 (2016).

218. Shao, X.*, et al.* CellTalkDB: a manually curated database of ligand–receptor interactions in humans and mice. *Briefings in bioinformatics* **22**, bbaa269 (2021).

219. Noël, F.*, et al.* Dissection of intercellular communication using the transcriptome-based framework ICELLNET. *Nature communications* **12**, 1-16 (2021).

220. Zhang, Y.*, et al.* Cellinker: a platform of ligand–receptor interactions for intercellular communication analysis. *Bioinformatics* **37**, 2025-2032 (2021).

221. Xu, C., Ma, D., Ding, Q., Zhou, Y. & Zheng, H.L. PlantPhoneDB: A manually curated pan‐plant database of ligand‐receptor pairs infers cell–cell communication. *Plant Biotechnology Journal* **20**, 2123-2134 (2022).

222. Gao, S., Feng, X., Wu, Z., Kajigaya, S. & Young, N.S. CellCallEXT: analysis of ligand–receptor and transcription factor activities in cell–cell communication of tumor immune microenvironment. *Cancers* **14**, 4957 (2022).

223. Jin, S.*, et al.* Inference and analysis of cell-cell communication using CellChat. *Nature communications* **12**, 1-20 (2021).

224. Henzinger, T.A., Horowitz, B. & Kirsch, C.M. Giotto: A time-triggered language for embedded programming. in *International Workshop on Embedded Software* 166-184 (Springer, 2001).

225. Cabello-Aguilar, S.*, et al.* SingleCellSignalR: inference of intercellular networks from single-cell transcriptomics. *Nucleic Acids Research* **48**, e55-e55 (2020).

226. Zhang, Y.*, et al.* CellCall: integrating paired ligand–receptor and transcription factor activities for cell–cell communication. *Nucleic Acids Research* **49**, 8520-8534 (2021).

227. Li, D.*, et al.* TraSig: inferring cell-cell interactions from pseudotime ordering of scRNA-Seq data. *Genome biology* **23**, 1-19 (2022).

228. Wang, Y.*, et al.* iTALK: an R package to characterize and illustrate intercellular communication. *BioRxiv*, 507871 (2019).

229. Tyler, S.R.*, et al.* PyMINEr finds gene and autocrine-paracrine networks from human islet scRNA-Seq. *Cell reports* **26**, 1951-1964. e1958 (2019).

230. Jiang, Q.*, et al.* DeepCCI: a deep learning framework for identifying cell-cell interactions from single-cell RNA sequencing data. *bioRxiv* (2022).

231. Browaeys, R., Saelens, W. & Saeys, Y. NicheNet: modeling intercellular communication by linking ligands to target genes. *Nature methods* **17**, 159-162 (2020).

232. Cang, Z. & Nie, Q. Inferring spatial and signaling relationships between cells from single cell transcriptomic data. *Nature communications* **11**, 1-13 (2020).

233. Choi, H.*, et al.* Transcriptome analysis of individual stromal cell populations identifies stroma-tumor crosstalk in mouse lung cancer model. *Cell reports* **10**, 1187-1201 (2015).

234. Wang, S., Karikomi, M., MacLean, A.L. & Nie, Q. Cell lineage and communication network inference via optimization for single-cell transcriptomics. *Nucleic acids research* **47**, e66-e66 (2019).

235. Hou, R., Denisenko, E., Ong, H.T., Ramilowski, J.A. & Forrest, A.R. Predicting cell-to-cell communication networks using NATMI. *Nature communications* **11**, 1-11 (2020).

236. Jakobsson, J.E., Spjuth, O. & Lagerström, M.C. scConnect: a method for exploratory analysis of cell–cell communication based on single-cell RNA-sequencing data. *Bioinformatics* **37**, 3501-3508 (2021).

237. Jung, S., Singh, K. & Del Sol, A. FunRes: resolving tissue-specific functional cell states based on a cell–cell communication network model. *Briefings in Bioinformatics* **22**, bbaa283 (2021).

238. Tsuyuzaki, K., Ishii, M. & Nikaido, I. Uncovering hypergraphs of cell-cell interaction from single cell RNA-sequencing data. *BioRxiv*, 566182 (2019).
